# Epigenetic mediators of diet and lifestyle and insulin resistance

**DOI:** 10.1186/s12263-025-00792-7

**Published:** 2026-01-24

**Authors:** Oladimeji J. Akinlawon, Laurence D. Parnell, Fang Liu, Donna K. Arnett, Jose M. Ordovas, Chao-Qiang Lai

**Affiliations:** 1 USDA Agricultural Research Service, Precision Nutrition Team, JM-USDA Human Nutrition Research Center on Aging at Tufts University, 711 Washington Street, Boston, MA 02111 USA; 2 Precision Nutrition Team, JM-USDA Human Nutrition Research Center on Aging at Tufts University, Boston, MA 02111 USA; 3 Office of the Provost, University of South Carolina, Columbia, SC 29208 USA; 4 IMDEA-Food Institute, CEI UAM + CSIC, Madrid, 28049 Spain; 5 CIBER Physiopathology of Obesity and Nutrition (CIBEROBN) Institute of Health Carlos III, Madrid, Spain

**Keywords:** Diet and lifestyle factors, DNA methylation, Epigenome-wide association study, Homeostasis model assessment for insulin resistance

## Abstract

**Background:**

Implementing personalized dietary interventions has become important as emerging evidence indicates that dietary and lifestyle-dependent epigenetic modifications affect insulin resistance.

**Methods:**

An epigenome-wide association study (EWAS) was conducted on 1,684 non-diabetic adults aged 57–75 from the Framingham Offspring Study (FOS). Associations between dietary and lifestyle factors (assessed via food frequency questionnaire) and DNA methylation sites (DNA-MS) were analyzed, with adjustments for confounders (age, sex, lifestyle) and multiple testing. Significant epigenetic mediators were evaluated using causal mediation models. Validation was executed using the Genetics of Lipid-Lowering Drugs and Diet Network Study (*n* = 945).

**Results:**

The EWAS identified 35 DNA-MS linked to HOMA-IR, with 13 DNA-MS showing significant associations with dietary factors in the FOS. Key DNA-MS including cg17901584 (*DHCR24*), influenced by brown rice (natural indirect effect: β ± SE, -0.02 ± 0.01, *p* = 0.0003), cg22761431 (*EFNB3*) by wheat germ (-0.01 ± 0.003, *p* = 0.001), and cg00574958 (*CPT1A*) associated with lactose (-0.001 ± 0.0003, *p* = 0.0001) intakes, all correlated with decreased HOMA-IR. Other DNA-MS, cg06808571 (*KCNH2*), cg06690548 (*SLC7A11*), and intergenic cg07504977, mediated increases in HOMA-IR linked to intakes of low-calorie cola (0.003 ± 0.001, *p* = 0.001), alcohol (0.01 ± 0.001, *p* = 9.0E-11), and palmitoleic acid (0.03 ± 0.01, *p* = 9.0E-05), respectively. In the GOLDN study, alcohol intake mediated by cg06690548 methylation in *SLC7A11* was positively associated with HOMA-IR. Notably, the *DHCR24* gene, crucial for cholesterol biosynthesis, was highlighted as a potential dietary target for reducing metabolic risk.

**Conclusion:**

The identification of specific DNA methylation sites, such as those in *DHCR24* and *EFNB3*, provides supportive evidence for the mechanistic basis of known dietary effects on metabolic health. These findings not only reinforce the importance of diet in managing insulin resistance but also open avenues for personalized dietary interventions tailored to an individual’s epigenetic profile.

**Supplementary Information:**

The online version contains supplementary material available at 10.1186/s12263-025-00792-7.

## Introduction

Insulin resistance (IR) is a key metabolic abnormality that is commonly associated with several chronic health conditions, including reduced levels of high-density lipoprotein cholesterol, hypertension, elevated triglycerides, and fasting glucose levels [1]. IR is considered a primary pathological mechanism in the development of type 2 diabetes, which affects approximately 9.3% of the global population [2]. Initially, the body compensates for the increased demand for insulin by enhancing β-cell function. However, prolonged insulin resistance can lead to β-cell failure, resulting in insufficient insulin production, elevated blood glucose levels, and ultimately the onset of type 2 diabetes or other metabolic diseases [1, 3].

IR is a complex condition influenced by both genetic and environmental factors, including lifestyle factors such as physical activity and unhealthy dietary patterns [4]. While genetic variation typically involves direct alterations in the DNA sequence, epigenetics refers to changes in gene expression and function that are inherited through cell divisions without modifications to the underlying DNA code [4]. Epigenetic processes, such as DNA methylation, histone modification, and non-coding RNA regulation, are important in regulating gene expression and can be influenced by various environmental exposures, including diet [4].

Epigenome-wide association studies (EWAS) have identified numerous epigenetic signatures linked to IR and other metabolic disorders [5, 6]. An extension of those basic EWAS results has identified that epigenetic modifications mediate the effects of diet and lifestyle on IR [4]. Research suggests that diets with health-promoting properties, such as low-calorie or nutrient-rich diets, may exert their beneficial effects through changes in the epigenome [7, 8]. Conversely, poor dietary patterns, such as high intake of refined carbohydrates or unhealthy fats, also can lead to epigenetic changes that exacerbate insulin resistance and related metabolic conditions [7, 8].

Our previous work demonstrated that specific dietary components, such as carbohydrate intake and the carbohydrate-to-fat ratio, were associated with altered DNA methylation patterns, including the *CPT1A* methylation site cg00574958 [9]. Other studies have linked diet-induced changes in epigenetic markers with improvements in insulin secretion and reductions in epigenetic age [10, 11]. Nutrients like glucose and palmitate, which influence insulin secretion, have been shown to alter DNA methylation in genes related to IR [12–14].

In addition to their effects on the individual, epigenetic modifications can be passed down through generations, potentially affecting the health of future offspring [15–17]. These findings emphasize the importance of understanding how diet and environmental factors influence the epigenome and the long-term risk of developing insulin resistance. Notably, there is growing evidence that some epigenetic modifications are reversible [4], suggesting that lifestyle interventions may mitigate the negative impact of poor dietary habits.

The goal of this study is to expand on our prior research [18] by conducting an epigenome-wide association analysis to identify specific epigenetic signatures that mediate the role of diet and lifestyle factors on Homeostasis Model Assessment for Insulin Resistance (HOMA-IR), in the Framingham Offspring Study (FOS). Subsequently, we aim to validate our findings in the Genetics of Lipid-Lowering Drugs and Diet Network (GOLDN) study. By identifying epigenetic markers associated with dietary and lifestyle factors, this research seeks to enhance our understanding of how environmental influences contribute to IR and provide insights for future educational programs and targeted interventions in specific populations.

## Study Populations and Methods

### The Framingham Offspring Study (FOS)

**Fig. 1:**
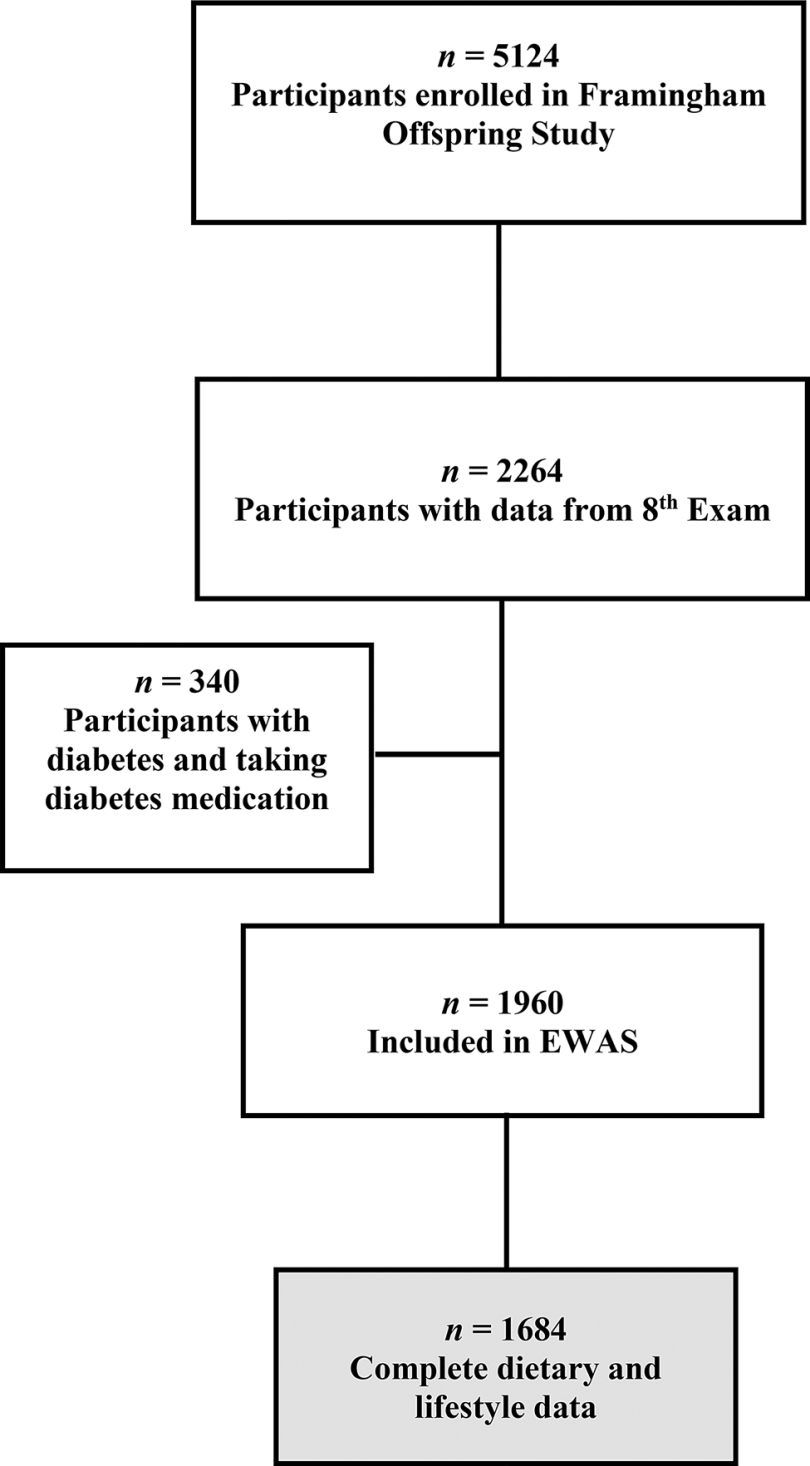
Flowchart of study design

The Framingham Heart Study (FHS) is a prospective longitudinal study of individuals of European descent residing in Framingham, MA. The study, launched in 1948, aimed to identify key factors linked with cardiovascular diseases [19]. In 1971, the recruitment of the Framingham offspring cohort began with 5124 participants enrolled (Fig. 1), consisting of the spouses and offspring of the parent cohort [19]. Participants were interviewed and physical and clinical examinations were conducted at intervals of 4–8 years. This study uses data from participants in the eighth exam of the FOS, aged 40–92 y, with complete diet and health information and with available whole-genome DNA methylation data. These data were requested via controlled access from dbGaP (https://dbgap.ncbi.nlm.nih.gov, with study accession phs000007.v25.p9, downloaded on 27 September 2017).

#### The Genetics Of Lipid-lowering Drugs and Diet Network study

The Genetics of Lipid-Lowering Drugs and Diet Network (GOLDN) study has been described in detail [20]. Briefly, the GOLDN study is a component of the National Institute of Health National Heart, Lung, and Blood Institute Family Heart Study. The study consists of 1,327 participants from families of European descent, aged 18 to 92 years with a mean age of 48.7 years, and recruited from two study centers: Minneapolis, MN, and Salt Lake City, UT. Information on demographics, lifestyle, and diet was collected using diet history questionnaires. The study protocol was approved by the Institutional Review Boards at Tufts University, the University of Minnesota, the University of Utah, and the University of Alabama at Birmingham, United States. Written consent was obtained from participants. A total of 945 participants with complete whole-genome DNA methylation and dietary data at baseline were included for analysis. The GOLDN cohort shares comparable European ancestry to FOS, thus making GOLDN an appropriate population for validating the findings from FOS. The data can be accessed from dbGaP (https://dbgap.ncbi.nlm.nih.gov) with study accession phs000741.v2.p1.

#### Homeostasis model assessment for insulin resistance

In FOS, blood samples were obtained following a 12-hour fasting period. Glucose levels were measured using hexokinase reagent, and insulin levels were determined using a radioimmunoassay technique for total immunoreactive insulin (Coat-A-Count Insulin, Diagnostic Products, Los Angeles, CA). The homeostasis model assessment for insulin resistance (HOMA-IR) was estimated as insulin (µU/mL) × [glucose (mg/dL) × 0.055]/22.5 as described [21]. The average intra-assay coefficient of variation for fasting glucose and insulin was 2% to 3%. Participants with diabetes who used diabetes medication were excluded to mitigate the potential distortion of HOMA-IR, primarily attributed to the influence of glucose levels. Normality was tested for HOMA-IR and was subsequently log-transformed due to its skewed distribution before further analysis.

In the GOLDN study, blood samples were collected after an overnight fast. Plasma glucose was measured using a Hitachi commercial kit (Roche Diagnostics) by a hexokinase mediated reaction. Plasma insulin was obtained using a competitive radioimmunoassay (Linco Research, St. Charles, MO, USA). Intra-laboratory reliability of glucose and insulin measurements were 0.984 and 0.975, respectively. HOMA-IR was estimated using the formula above.

#### Genome-wide DNA methylation

As described [22, 23], and with wording partly reproduced here, genome-wide DNA methylation of isolated DNA samples in both cohorts (FOS and GOLDN) were analyzed using Infinium HumanMethylation450 K arrays, Illumina. DNA methylation data for FOS were obtained from dbGaP (accession phs000724.v9.p13), and quality control processing was conducted on the raw IDAT files for both study populations. Principal components (PCs) were estimated from β scores of the altered autosomal DNA methylation sites (DNA-MS) to account for heterogenous blood cell-type composition among the samples, using the PCA function applied in SNP and Variation Suite (SVS 8.9.0, Golden Helix Inc., Bozeman, MT). Similar to prior studies [5, 24], the first five PCs were used as covariates in all assessments to control for the variability of distinct cell types. A total of 415,202 DNA-MS was retained after quality control and included in this study. Of these 76.7% were annotated as genic and the remaining were categorized as intergenic across the genome. Annotation was conducted using the human genome build GRCh38/hg38.

#### Dietary and lifestyle factors

The FOS dietary data were assessed from a 126-item Willett semi-quantitative food frequency questionnaire (FFQ), which was adapted for the study population. This questionnaire captured detailed information on daily nutrient intake, including macronutrients, minerals, vitamins, fiber, bioactives, and food groups [25]. Physical activity levels were assessed using a modified version of the Harvard Alumni Paffenbarger questionnaire [25]. Other lifestyle factors, such as alcohol consumption (in grams per day) and smoking (number of cigarettes per day), were also collected. For the GOLDN cohort, dietary intake was assessed using the Diet History Questionnaire, which was based on the Harvard University Food Composition Database, the USDA database, and the Minnesota Nutrient System. These data allowed for the assessment of a wide range of dietary exposures and lifestyle factors across both study populations.

### Statistical analysis

#### Epigenome-wide Association Study (EWAS)

To identify the DNA-MS associated with HOMA-IR in FOS, an epigenome-wide association study (EWAS) was conducted using a linear mixed model [26]. Continuously expressed log-transformed HOMA-IR (lgHOMA-IR) was regressed on the DNA-MS. The model was adjusted for potential confounding factors, including age, sex, physical activity, smoking, and cell-type heterogeneity, with kinship included as a random effect. Bonferroni correction was applied to adjust for multiple testing, establishing an epigenome-wide significance threshold at 1.10E-7 [26]. To estimate the proportion of variance in lgHOMA-IR explained by the identified epigenetic loci, a multi-locus mixed model was used. This model was similarly adjusted for the same covariates (age, sex, physical activity, smoking, cell-type heterogeneity, and kinship) and applied exclusively to non-diabetic participants to mitigate any confounding effect from diabetes. Analyses were conducted using SVS 8.9.0 (Bozeman, MT).

#### Association between dietary and lifestyle factors and HOMA-IR in FOS

To examine the associations between dietary and lifestyle (DL) factors and DNA-MS identified in relation to lgHOMA-IR in FOS, regression analyses were performed for each DNA-MS on the 392 DL factors. Models were adjusted for age, sex, physical activity, smoking, total energy intake, and cell-type heterogeneity, with kinship included as a random effect to account for familial relationships. To correct for multiple testing, correlation matrix analysis was conducted using R programming language (R version 4.2.2, R Core Team, 2022) to estimate the number of independent DL factors. Bonferroni test was applied to account for multiple testing across the number of independent DL factor clusters, setting the significance threshold accordingly.

#### Mediation analysis

In FOS, for each DNA-MS and associated DL factors, one representative diet/lifestyle factor was selected within each classified cluster of DL factors based on the lowest *p*-value and was processed with the corresponding DNA-MS for mediation analysis. Bonferroni adjustment was applied to the total number of independent DL factors associated with a lgHOMA-IR-associated DNA-MS and was used to determine the significance threshold for multiple tests for the mediation analysis. Causal mediation analysis was conducted using the CAUSALMED procedure in Statistical Analysis System (SAS) statistical software (SAS version 9.4; SAS Institute Inc., Cary, NC, USA). The total effect (TE), natural direct effect (NDE), and natural indirect effect (NIE) were estimated adjusting for age, sex, physical activity, smoking, total energy intake, and cell-type heterogeneity. The NIE estimated the effect of each dietary and lifestyle factor on HOMA-IR mediated by the DNA-MS, while NDE estimated the residual effect not mediated by the DNA-MS. The TE is the sum of both direct and indirect effects. Correlation matrix analysis was conducted as above for the DNA-MS, thereby classifying and determining the number of independent DNA-MS. The most significantly associated DNA-MS with its corresponding DL factor was then selected from each DNA-MS cluster based on NIE *p*-value, as a top mediator.

#### Validation study using the GOLDN study

For validation, DNA-MS associated with lgHOMA-IR in the FOS were identified in the GOLDN study. We examined the association between the identified DNA-MS with DL factors in a linear mixed model adjusting for age, sex, physical activity, smoking, total energy intake, cell-type heterogeneity, center, and kinship. Finally, mediation analyses were conducted for selected DNA-MS similar to those in FOS. The significance threshold was defined as *p* ≤ 0.05.

## Results

### Demographic, lifestyle, biochemical, and dietary characteristics

Table 1 presents the demographic, lifestyle, biochemical, and dietary characteristics of the two independent cohorts, the FOS and the GOLDN Study. T-tests and chi-square tests were used to examine differences between means and categories, respectively. Participants in FOS were older (Mean ± SD, 65.9 ± 8.87 vs. 47.9 ± 16.4 years, *P* < 0.0001) compared to those in GOLDN. FOS participants also had a lower prevalence of current smokers (7.36% vs. 27.8%, *P* < 0.0001) and were more physically active (35.5 ± 5.35 vs. 34.3 ± 6.37, *P* < 0.0001) than those in the GOLDN cohort. Biochemically, FOS participants had higher glucose concentrations (101 ± 9.42 mg/dL vs. 99.0 ± 12.8 mg/dL, *P* < 0.001) but lower insulin levels (10.0 ± 6.94 mU/L vs. 13.5 ± 7.79 mU/L, *P* < 0.0001) and HOMA-IR measured as insulin resistance (2.56 ± 1.96 vs. 3.39 ± 2.29, *P* < 0.0001) compared to GOLDN participants.

**Table 1:** Characteristics of the study population^1^

	FOS	GOLDN	*P* ^2^
n	1684	945	
Men/women, n	725/959	449/496	0.03
Age, y	65.9 ± 8.87	47.9 ± 16.4	< 0.0001
Current smoker, %			
No	92.6	72.2	
Yes	7.36	27.8	< 0.0001
	Mean ± SD	Mean ± SD	
Physical activity score	35.5 ± 5.35	34.3 ± 6.37	< 0.0001
Glucose, mg/dl	101 ± 9.42	99.0 ± 12.8	< 0.001
Insulin, mU/L	10.0 ± 6.94	13.5 ± 7.79	< 0.0001
Insulin resistance (HOMA_IR)	2.56 ± 1.96	3.39 ± 2.29	< 0.0001
Carbohydrate intake (% of total energy)	46.9 ± 8.70	49.1 ± 8.54	< 0.0001
Total fat intake (% of total energy)	32.7 ± 6.52	35.4 ± 6.71	< 0.0001
MUFAs (% of total energy)	12.3 ± 2.80	13.3 ± 2.79	< 0.0001
PUFAs (% of total energy)	6.26 ± 1.77	7.64 ± 2.12	< 0.0001
Total energy intake, kcal	1885 ± 625	2133 ± 1213	< 0.0001

^1^Includes participants without evidence of diabetes

^2^*P* difference in population means (t-test) or between categories (Chi-square test)

Dietary data further highlighted differences between the two cohorts. FOS participants had lower percentages of total energy intake from carbohydrates (46.9% vs. 49.1%, *P* < 0.0001), total fat (32.7% vs. 35.4%, *P* < 0.0001), monounsaturated fatty acids (12.3% vs. 13.3%, *P* < 0.0001), and polyunsaturated fatty acids (6.26% vs. 7.64%, *P* < 0.0001) compared to GOLDN participants. Additionally, the total energy intake was lower in FOS (1885 ± 625 kcal vs. 2133 ± 1213 kcal, *P* < 0.0001) compared to GOLDN participants.

### Epigenome-wide association study (EWAS) in the FOS

After adjusting for covariates in linear mixed model analyses, we identified 35 DNA-MS significantly associated with log-transformed HOMA-IR (lgHOMA-IR) in the FOS cohort (*p* < 1.1 × 10^-7) (Table 2). Of these, several DNA-MS explained a significant proportion of the variance in lgHOMA-IR. The DNA-MS positively associated with HOMA-IR with the largest phenotypic variance were cg06500161 in the *ABCG1* gene (β ± SE, 4.16 ± 0.36, *p* = 1.23E-29) with 6.35% phenotypic variance, cg11024682 in the *SREBF1* gene (2.83 ± 0.38, *p* = 9.04E-14) with 2.81%, and cg07504977 (2.19 ± 0.30, *p* = 3.64E-13) with 2.68%. The cg17901584 in the *DHCR24* gene (-2.43 ± 0.25, *p* = 3.76E-21) with 4.47%, cg00574958 in the *CPT1A* gene (-7.07 ± 0.82, *p* = 8.28E-18) with 3.72%, cg17501210 in the *RPS6KA2* gene (-1.94 ± 0.27, *p* = 4.15E-13) with 2.66%, and cg17058475 in the CPT1A gene (-4.32 ± 0.62, *p* = 3.20E-12) with 2.46% were inversely associated with HOMA-IR.

**Table 2:** Epigenetic markers associated with homeostatic model assessment for insulin resistance in the 8th exam Framingham offspring study (*n* = 1958)

DNA-MS	Chromosome	Position GRCh37/hg19	Gene	β	SE	Variance explained (%)	*P* value
cg17901584	1	55,353,706	DHCR24	-2.4263	0.2541	4.47	3.76E-21
cg14476101	1	120,255,992	PHGDH	-1.2121	0.1961	1.92	7.79E-10
cg12593793	1	156,074,135	NA	-2.6699	0.4629	1.68	9.35E-09
cg25001190	1	61,668,835	NFIA	-1.4657	0.2677	1.51	4.93E-08
cg25217710	1	156,609,523	NA	2.4377	0.4492	1.49	6.44E-08
cg12620005	1	51,779,655	TTC39A	-2.9396	0.5424	1.48	6.70E-08
cg03165356	2	216,882,347	NA	-1.4491	0.2582	1.59	2.30E-08
cg25758828	2	113,976,143	PAX8;LOC440839	-2.1705	0.3932	1.54	3.83E-08
cg06690548	4	139,162,808	SLC7A11	-1.2659	0.1864	2.31	1.45E-11
cg26403843	5	158,634,085	RNF145	1.4923	0.2337	2.05	2.14E-10
cg17501210	6	166,970,252	RPS6KA2	-1.9362	0.2652	2.66	4.15E-13
cg18120259	6	43,894,639	LOC100132354	-2.1781	0.3579	1.87	1.39E-09
cg13123009	6	31,681,882	LY6G6E; LY6G6D	2.3198	0.4255	1.50	5.61E-08
cg21429551	7	30,635,762	GARS	-1.2377	0.1918	2.09	1.36E-10
cg06808571	7	150,642,256	KCNH2	2.8205	0.4462	2.01	3.20E-10
cg19390658	7	30,636,176	GARS	-1.2078	0.2262	1.44	1.05E-07
cg13847322	9	137,272,766	RXRA	1.8581	0.3431	1.48	6.83E-08
cg07504977	10	102,131,012	NA	2.1910	0.2993	2.68	3.638E-13
cg26572392	10	24,496,943	KIAA1217	1.9183	0.3331	1.67	9.82E-09
cg03290131	10	112,263,831	DUSP5	-2.0735	0.3834	1.48	7.16E-08
cg00574958	11	68,607,622	CPT1A	-7.0742	0.8151	3.72	8.28E-18
cg17058475	11	68,607,737	CPT1A	-4.3193	0.6159	2.46	3.20E-12
cg06192883	15	52,554,171	MYO5C	2.22411	0.3502	2.03	2.66E-10
cg20507228	15	91,460,071	MAN2A2	1.5916	0.2553	1.96	5.55E-10
cg03500056	16	8,814,507	ABAT	2.9475	0.4273	2.38	7.07E-12
cg00472758	16	2,552,820	TBC1D24	2.5798	0.4143	1.95	5.83E-10
cg09581649	16	4,572,735	C16orf5	2.8461	0.5137	1.55	3.42E-08
cg11024682	17	17,730,094	SREBF1	2.8349	0.3775	2.81	9.04E-14
cg09664445	17	2,612,406	KIAA0664	3.1362	0.4970	2.00	3.45E-10
cg22761431	17	7,609,416	EFNB3	2.0326	0.3581	1.63	1.59E-08
cg07952905	17	27,275,352	PHF12	2.5780	0.4832	1.44	1.06E-07
cg06500161	21	43,656,587	ABCG1	4.1608	0.3620	6.35	1.23E-29
cg27243685	21	43,642,366	ABCG1	2.4863	0.4017	1.93	7.34E-10
cg08309687	21	35,320,596	NA	-1.2719	0.2289	1.56	3.13E-08
cg09349128	22	50,327,986	NA	-2.5951	0.4595	1.61	1.86E-08

Model adjusted for age, sex, physical activity, smoking, cell-type heterogeneity, and kinship

### Association between DNA-MS and dietary lifestyle factors

**Fig. 2:**
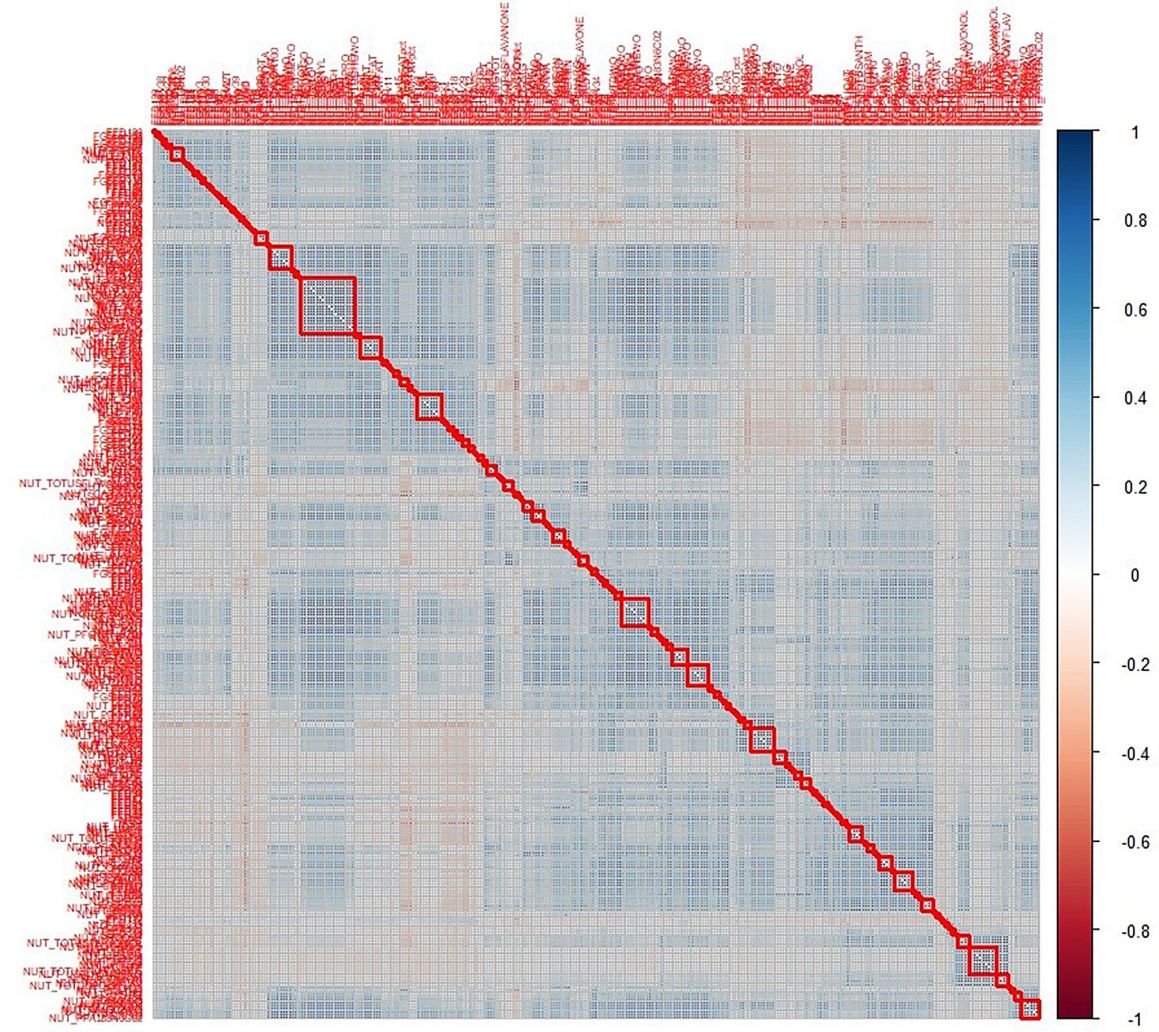
Correlation plot identifying 170 clusters from 392 dietary and lifestyle factors

Following the EWAS analysis, we examined the relationship between the 35 identified DNA-MS for lgHOMA-IR and dietary lifestyle (DL) factors. After adjusting for potential confounding variables, 18 DNA-MS were found to have statistically significant associations with at least one dietary factor (Supplemental Table 1). To account for multiple testing, a significance threshold of *p* < 0.0003 (*p* < 0.05/170) was applied, after identifying that 170 clusters of DL factors describe the relationships among and between the DL factors. This information is presented in a correlation matrix (Fig. 2). Among these DNA-MS, 13 had a significant association with at least one representative DL factor, with the strongest associations from the clusters of DL factors (Table 3). In total, 35 DL factors were found to be associated with the DNA-MS linked to lgHOMA-IR, setting the significance threshold for the natural indirect effect (NIE) mediation analysis at *p* < 0.0014 (*p* < 0.05/35) (Supplemental Table 2). No DL factor that was identified as a component of a significant association with lgHOMA-IR appeared in more than one such association (Table 3).

**Table 3:** Dietary or lifestyle factors associated with homeostatic model assessment for insulin Resistance-associated epigenetic signatures in the 8th exam Framingham offspring study (*n* = 1655–1671)

DNA-MS	Dietary/lifestyle	β	SE	Variance Explained (%)	*P* value
cg07504977	c9,t11 conjugated diene isomer 18:2 Linoleic acid	0.0001	2.9E-05	1.17	1.04E-05
cg14476101	Dihydrovitamin K	0.0006	0.0002	0.85	1.73E-04
cg07504977	Palmitic fatty acid	0.0017	0.0004	1.18	9.03E-06
cg00574958	Total fat (energy adjusted)	-0.0003	6.7E-05	1.16	1.10E-05
cg06192883	Eicosapentaenoic fatty acid (EPA)	-0.0264	0.0069	0.88	1.27E-04
cg00574958	Phosphorous	8.2E-06	1.8E-06	1.23	5.87E-06
cg06690548	Alcohol servings	-0.0037	0.0002	14.4	3.39E-58
cg06690548	Manganese without supplements	0.0083	0.0017	1.38	1.52E-06
cg07504977	Cholesterol	5.9E-05	1.2E-05	1.40	1.36E-06
cg00574958	Thiamine B1 without vitamin pills	0.0054	0.0010	1.62	1.91E-07
cg06690548	Free Choline without supplements	-0.0007	0.0001	1.80	4.09E-08
cg06690548	Natural Bran	0.0020	0.0005	1.19	8.41E-06
cg17501210	Calcium	1.2E-05	2.6E-06	1.35	2.11E-06
cg00574958	Lactose	0.0002	3.9E-05	1.57	3.11E-07
cg06690548	Sucrose	0.0007	0.0001	3.04	8.07E-13
cg07504977	Palmitoleic fatty acid	0.0157	0.0027	2.01	6.74E-09
cg17058475	Natural food folate	2.1E-05	5.7E-06	0.84	1.85E-04
cg22761431	Wheat germ	-0.0044	0.0009	1.28	3.79E-06
cg00574958	Vitamin D without vitamin pills	1.1E-05	2.9E-06	0.80	2.69 E-04
cg06690548	Red wine	-0.0039	0.0005	3.65	4.23E-15
cg06690548	Catechin	-0.0010	0.0002	1.70	9.66E-08
cg06690548	Apigenin	-0.0083	0.0015	1.73	7.55E-08
cg07504977	Animal fat (energy adjusted)	0.0010	0.0002	1.48	6.81E-07
cg06690548	Carbohydrate (energy adjusted)	0.0018	0.0002	3.62	5.33E-15
cg00574958	Butter	-0.0003	8.8E-05	0.94	7.30E-05
cg07504977	Processed Meat servings	0.0017	0.0004	0.95	6.89E-05
cg09581649	Liver	-0.0231	0.0061	0.85	1.61E-04
cg17901584	Brown rice	0.0082	0.0020	1.06	2.73E-05
cg06808571	Low calorie cola, no caffeine	0.0010	0.0002	1.04	3.19E-05
cg06690548	White wine	-0.0051	0.0006	4.35	9.14E-18
cg06690548	Liquor	-0.0045	0.0005	4.87	8.98E-20
cg06690548	Sweet baked goods servings	0.0017	0.0003	1.50	5.41E-07
cg18120259	Mustard	-0.0023	0.0006	0.91	1.01E-04
cg00574958	DASH diet	0.0003	8.4E-05	0.92	9.20E-05
cg12593793	Jams/jellies	0.0009	0.0003	0.81	2.37E-04

Model adjusted for age, sex, physical activity, smoking, total energy intake, cell-type heterogeneity, and kinship

### Mediation analysis of dietary lifestyle factors and epigenetic modifiers

**Fig. 3:**
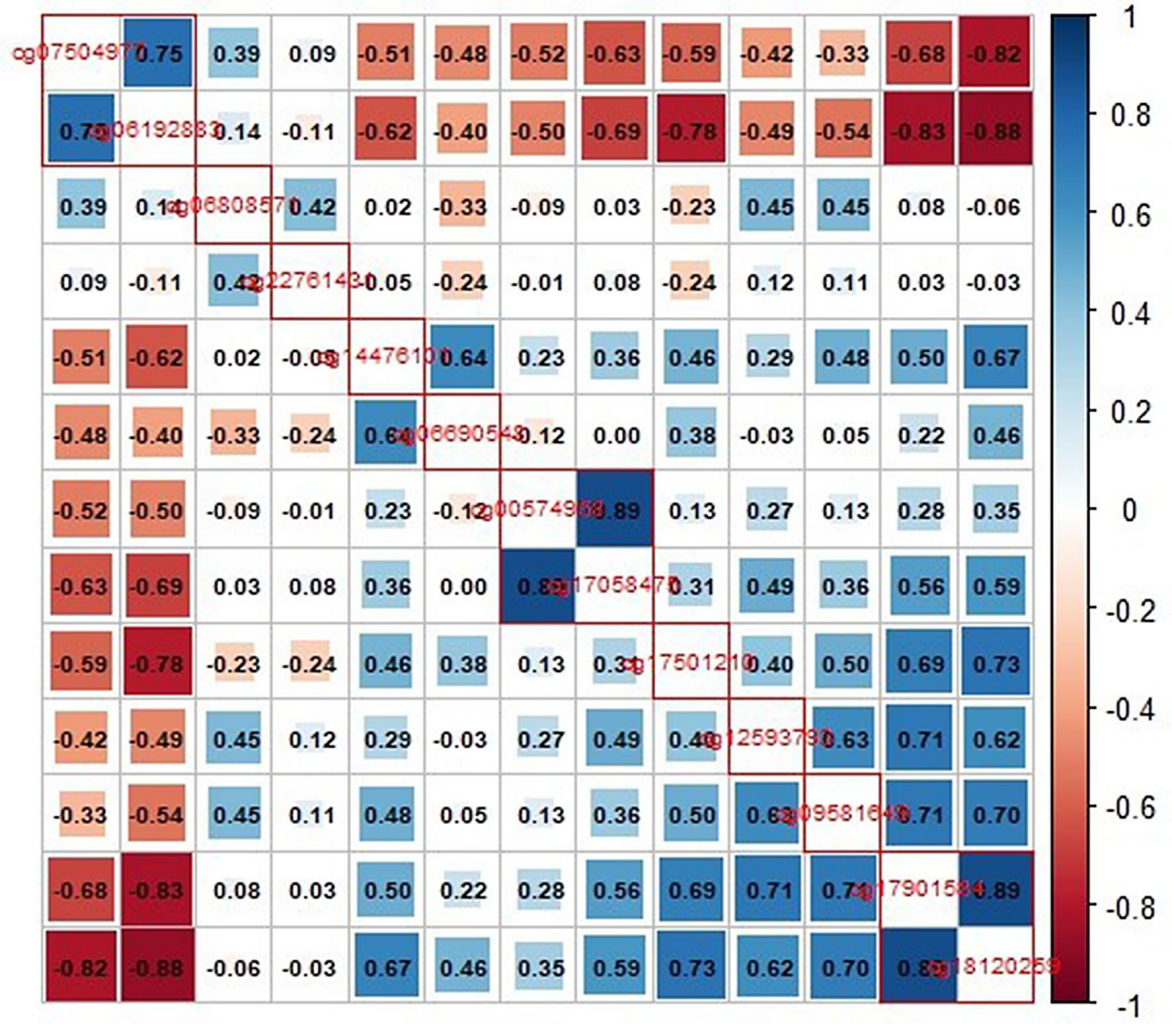
Correlation plot identifying 10 clusters from 13 DNA methylation sites

To identify the top mediators, a correlation matrix was conducted for the 13 DNA-MS, revealing 10 clusters (Fig. 3 and Supplemental Table 2). Key DNA-MS were selected as top mediators based on the strength of correlation with the associated dietary factor (Table 4). In the FOS cohort, significant inverse associations were observed between lgHOMA-IR and intakes of brown rice mediated by cg17901584 methylation in *DHCR24* (NIE: β ± SE, -0.02 ± 0.01, *p* = 0.0003), wheat germ mediated by cg22761431 in *EFNB3* (-0.01 ± 0.003, *p* = 0.001), and lactose mediated by cg00574958 in *CPT1A* (-0.001 ± 0.0003, *p* = 0.0001). Conversely, significant positive associations were observed between lgHOMA-IR and intakes of alcohol mediated by cg06690548 methylation in *SLC7A11* (0.01 ± 0.001, *p* = 9.0E-11), low-calorie cola mediated by cg06808571 in *KCNH2* (0.003 ± 0.001, *p* = 0.001), and palmitoleic acid mediated by intergenic cg07504977 (0.03 ± 0.01, *p* = 9.0E-05).

**Table 4:** Indirect effects of dietary/lifestyle factors on homeostatic model assessment for insulin resistance through epigenetic signatures in the 8th exam Framingham offspring study

DNA-MS	Chromosome	Position	Genes	Dietary/lifestyle	β	SE	*P* value
cg17901584	1	55,353,706	DHCR24	Brown rice	-0.0208	0.0057	0.0003
cg14476101	1	120,255,992	PHGDH	Dihydrovitamin K	-0.0008	0.0002	0.001
cg06690548	4	139,162,808	SLC7A11	Alcohol servings	0.0058	0.0009	9.0E-11
cg06808571	7	150,642,256	KCNH2	Low calorie cola, no caffeine	0.0026	0.0008	0.0012
cg07504977	10	102,131,012	NA	Palmitoleic fatty acid	0.0331	0.0085	9.0E-05
cg00574958	11	68,607,622	CPT1A	Lactose	-0.0011	0.0003	0.0001
cg22761431	17	7,609,416	EFNB3	Wheat germ	-0.0086	0.0025	0.0007

Model adjusted for age, sex, physical activity, smoking, total energy intake, cell-type heterogeneity, and kinship

### Replication analysis in the GOLDN study

In the GOLDN cohort, we evaluated the DNA-MS similarly associated with those identified in the FOS cohort with lgHOMA-IR. Of the 35 DNA-MS associated with lgHOMA-IR in FOS, 20 were significantly associated with lgHOMA-IR in GOLDN (*p* < 0.05) (Supplemental Table 3). The DL factors in GOLDN primarily are described as nutrients and therefore provided little direct comparison to the DL factors in FOS, which consist of nutrients, foods and food groups. After conducting mediation analysis based on the identified DNA-MS and DL factors with significant NIE in the FOS, we found only energy-adjusted alcohol intake was similarly positively significantly associated with lgHOMA-IR in both cohorts. DL factors associated with the DNA-MS in the GOLDN cohort are provided in Supplemental Table 4. This association was mediated by cg06690548 methylation in *SLC7A11* (0.002 ± 0.001, *p* = 0.01), which mirrored the results observed in the FOS cohort (Supplemental Table 5).

## Discussion

Our analysis highlights DNA methylation signatures that mediate the association between dietary factors and HOMA-IR at a suggestive significance level in the Framingham Offspring Study. Intakes of brown rice, wheat germ, and lactose were inversely associated with HOMA-IR, with their effects significantly mediated by cg17901584 – *DHCR24*, cg22761431 – *EFNB3*, and cg00574958 – *CPT1A*, respectively. Brown rice and wheat germ are both rich in fiber and polyphenols which are known for their role in moderating glycemic levels and altering DNA methylation patterns [27–29]. Our findings support previous research suggesting that high fiber diets, particularly those with a low glycemic index, can reduce insulin resistance by promoting slower glucose absorption [30].

The genes that contain the three primary DNA methylation sites have established functions relating to energy and glucose homeostasis. DHCR24 has been associated with oxidative stress regulation and insulin signaling [31]. Increased brown rice intake may lower HOMA-IR levels via methylation at cg17901584 in the *DHCR24* gene by altering cholesterol membrane patterns and decrease oxidative stress, thereby enhancing insulin responsiveness [32]. EFNB3 is involved in energy balance and glucose metabolism [33]. High glycemic diets are linked to increased DNA methylation patterns that impair insulin secretion [12, 13], further supporting the idea that dietary choices can have lasting molecular effects on insulin regulation. Wheat germ contains methyl-donor nutrients such as butaine and choline which may contribute to site-specific methylation capacity at cg22761431 in *EFNB3* [34], thus, increase insulin sensitivity levels.

The inverse relationship between lactose and HOMA-IR is noteworthy, and the functions of lactate are important to consider. In short, lactose is a dietary component while lactate is glycolysis byproduct, circulating fuel, redox buffer and signaling molecule [35, 36]. Lactose, reflecting primarily milk and dairy product intake but also a component of many processed foods, facilitates the absorption of calcium and magnesium, minerals important for glucose homeostasis [37]. Biochemical metabolism of ingested lactose follows two paths - digestion of the disaccharide in the small intestine to glucose and galactose followed by absorption, or fermentation in the colon to lactate (lactic acid), then conversion to short-chain fatty acids (SCFAs) such as butyrate [36]. The first path sees the six-carbon sugars converted to pyruvate and ATP under aerobic conditions, while anaerobic conditions produce lactate. Elevated levels of lactate in blood are an indicator of insulin resistance and reduced oxidative capacity [38, 39]. Importantly, the SCFAs produced in the colon (the second path) have been shown to improve metabolic efficiency and insulin sensitivity, and more generally lactose has been shown to be a prebiotic that promotes health by modifying the gut microbiota profile (prompting the growth of beneficial bacteria) and increasing total SCFAs [40, 41]. Lactose also enhances fatty acid oxidation and has epigenetic regulatory roles associated with insulin signaling, as observed with the genetic variants of the lactase gene being associated with variations in insulin resistance in menopausal obese women [42]. In addition, reduced methylation level at cg00574958 of *CPT1A* has been linked with elevated fatty acid oxidation and increased insulin sensitivity [9]. As a result, habitual lactose intake may drive hypomethylation of *CPT1A* in this locus, thereby lowering HOMA-IR levels. This evidence suggests that lactose could have a positive impact on insulin sensitivity.

Further, we found that some diets inhibit reduction in HOMA-IR levels with notable patterns in DNA methylation modifications. Intakes of low-calorie cola, alcohol, and palmitoleic acid were positively associated with HOMA-IR, with their effects mediated by DNA methylation at cg06808571 – *KCNH2*, cg06690548 - *SLC7A11*, and intergenic cg07504977, respectively. Potassium channels, including *KCNH2* gene has influence on the pancreas and is involved in neuronal control of appetite and energy balance, glucose sensing, and insulin secretion [43]. Low-calorie cola contains artificial sweeteners, which have been shown to affect glucose tolerance and insulin sensitivity [44]. Metabolites derived from artificial sweeteners can trigger oxidative stress and one-carbon metabolism, resulting in DNA methylation changes, which may be pronounced in the methylation of Cg06808571 in *KCNH2*.

Alcohol consumption, particularly moderate intake, has been shown to exacerbate insulin resistance [45]. The impact of alcohol on DNA methylation is well-documented, with numerous studies linking genome-wide alterations in DNA methylation to alcohol intake [46]. Current evidence suggests association between DNA methylation at cg06690548 in *SLC7A11* with inflammation, metabolic stress and cardiometabolic disease risk [47]. Reduced methylation levels at cg06690548 - *SLC7A11* have been linked with heavy drinking days [18]. Although the antioxidant capacity of alcohol may be considered beneficial, prolonged exposure results in glutamate dysregulation, sustained oxidative stress and redox imbalance [48, 49].

The role of palmitoleic acid in insulin resistance remains unclear, with some studies suggesting no significant effect of monounsaturated fats on insulin sensitivity in diabetic individuals [50]. Our study suggests that epigenetic factors may modulate the relationship between palmitoleic acid and insulin sensitivity, offering a new avenue for exploration. The epigenetic signal at intergenic cg07504977 may affect palmitoleic acid concentration in tissues by modulating enzymes responsible for fatty acid desaturation and elongation [51], as the methylation site resides ~ 16.5 kb upstream of *SCD*, which encodes stearoyl-CoA desaturase. Further studies are needed to detail the relationship between cg07504977, SCD activity and insulin resistance.

### Strengths and limitations

The strengths of our study include the robust FOS dataset, which allowed for the identification of multiple epigenetic signatures linked to dietary factors and HOMA-IR. However, it is important to note that this study has several limitations. Study participants were predominantly of European descents living at Framingham, MA, thus, findings may not be generalized to other populations. Additionally, while DNA methylation was assessed in PBMCs, which are commonly used in epigenetic studies, they may not capture the full range of epigenetic signals associated with dietary lifestyle habits. Another limitation is the cross-sectional design of the study, which prevents us from establishing causal relationships between dietary factors, DNA methylation, and HOMA-IR. Also, the GOLDN study population, which was used for comparison, has a more limited set of dietary factors, reducing the ability to validate the FOS findings comprehensively. Future longitudinal studies are necessary to confirm these associations and determine how long-term adherence to specific dietary patterns affects epigenetic modifications and insulin resistance.

### Implications for future research

The results of this study have implications for future research on insulin resistance and epigenetics. The identification of specific DNA methylation sites associated with dietary habits offers potential targets for predictive models, such as machine learning algorithms, that could be used for early diagnosis or prevention of insulin resistance and related conditions. Additional research is needed to explore how adherence to these dietary factors impacts DNA methylation over time and investigate the underlying mechanisms through which epigenetic modifications influence insulin sensitivity.

## Conclusion

Our findings show the significant role of epigenetic modifications in mediating the effects of dietary factors on HOMA-IR. By understanding how these dietary components interact with the genome, deeper understanding of the molecular basis of insulin resistance can be acquired and more targeted personalized dietary interventions to prevent or manage insulin resistance based on individual epigenetic information can be developed. Future studies should further investigate the potential for epigenetic mapping to uncover additional environmental influences on insulin resistance and other metabolic disorders, offering new avenues for both prevention and treatment.

## Supplementary Information

Below is the link to the electronic supplementary material.

Supplementary Material 1

## Data Availability

Data used in this study are controlled access data. Data request should be directed to dbGaP (https://dbgap.ncbi.nlm.nih.gov) under accession numbers with study accession phs000007.v25.p9, phs000741.v2.p1.

## Abbreviations

DL factors: Diet and lifestyle factors
DNA-MS: DNA methylation site
EWAS: Epigenome-wide association study
FFQ: Food frequency questionnaire
FHS: Framingham Heart Study
FOS: Framingham offspring cohort
GOLDN: Genetics of Lipid-Lowering Drugs and Diet Network
HOMA-IR: Homeostasis model assessment for insulin resistance
IR: Insulin resistance
NDE: Natural direct effect
NIE: Natural indirect effect
SVS: SNP and VARIATION SUITE
TE: Total effect
