# Supplementary material for: Epigenetic mediators of diet and lifestyle and insulin resistance
Source: Genes Nutr. 2026 Jan 24;21:2. doi: 10.1186/s12263-025-00792-7 (PMC12914899; doi:10.1186/s12263-025-00792-7)
Supplement: Supplementary file 1 — Supplementary Material 1 [file 12263_2025_792_MOESM1_ESM.docx]

Supplementary Table 1. Dietary and lifestyle factors associated with Homeostatic Model Assessment for Insulin Resistance-associated epigenetic signatures in the Framingham Offspring Study (n = 1655 - 1671)

| **Dietary/lifestyle** | **Diet/lifestyle cluster** | DNA-MS | **Chromosome** | **Position GRCh37/hg19** | **Gene** | **β** | **SE** | **Variance Explained (%)** | **P value** |
| --- | --- | --- | --- | --- | --- | --- | --- | --- | --- |
| Animal fat | 1 | cg07504977 | 10 | 102131012 | NA | 0.0005 | 9.50E-05 | 1.8 | 3.30E-08 |
| c9,t11 conjugated diene isomer 18:2 Linoleic | 1 | cg07504977 | 10 | 102131012 | NA | 0.0001 | 2.90E-05 | 1.2 | 1.00E-05 |
| Animal fat | 1 | cg00574958 | 11 | 68607622 | CPT1A | -0.0001 | 3.50E-05 | 1 | 5.90E-05 |
| Dihydovitamin K | 2 | cg14476101 | 1 | 120255992 | PHGDH | 0.0006 | 0.0002 | 0.8 | 0.0002 |
| Palmitic fatty acid | 4 | cg07504977 | 10 | 102131012 | NA | 0.0017 | 0.0004 | 1.2 | 9.00E-06 |
| Palmitic fatty acid | 4 | cg00574958 | 11 | 68607622 | CPT1A | -0.0006 | 0.0001 | 1.2 | 1.00E-05 |
| Total Fat | 4 | cg00574958 | 11 | 68607622 | CPT1A | -0.0001 | 3.00E-05 | 1 | 3.60E-05 |
| Monounsaturated fat | 4 | cg00574958 | 11 | 68607622 | CPT1A | -0.0003 | 7.00E-05 | 0.9 | 7.20E-05 |
| Oleic | 4 | cg00574958 | 11 | 68607622 | CPT1A | -0.0003 | 7.50E-05 | 0.9 | 7.90E-05 |
| Saturated fat | 4 | cg07504977 | 10 | 102131012 | NA | 0.0008 | 0.0002 | 0.9 | 0.0001 |
| Saturated fat | 4 | cg00574958 | 11 | 68607622 | CPT1A | -0.0003 | 0.0001 | 0.8 | 0.0002 |
| Vegetable fat | 4 | cg06690548 | 4 | 139162808 | SLC7A11 | 0.0006 | 0.0002 | 0.8 | 0.0003 |
| Total fat (energy adjusted) | 5 | cg00574958 | 11 | 68607622 | CPT1A | -0.0003 | 0.0001 | 1.2 | 1.10E-05 |
| Total fat (energy adjusted) | 5 | cg00574958 | 11 | 68607622 | CPT1A | -0.0003 | 0.0001 | 1.2 | 1.10E-05 |
| Monounsaturated fat (energy adjusted) | 5 | cg00574958 | 11 | 68607622 | CPT1A | -0.0007 | 0.0002 | 1.1 | 1.90E-05 |
| Eicosapentaenoic fatty acid (EPA) | 6 | cg06192883 | 15 | 52554171 | MYO5C | -0.0264 | 0.0069 | 0.9 | 0.0001 |
| Phosphorous | 9 | cg00574958 | 11 | 68607622 | CPT1A | 8.20E-06 | 1.80E-06 | 1.2 | 5.90E-06 |
| Phosphorous without supplements | 9 | cg00574958 | 11 | 68607622 | CPT1A | 8.20E-06 | 1.80E-06 | 1.2 | 8.10E-06 |
| Cystine | 9 | cg06690548 | 4 | 139162808 | SLC7A11 | 0.0306 | 0.0072 | 1.1 | 2.20E-05 |
| Phosphorous without supplements | 9 | cg06690548 | 4 | 139162808 | SLC7A11 | 3.00E-05 | 8.10E-06 | 0.8 | 0.0002 |
| Phosphorous | 9 | cg17058475 | 11 | 68607737 | CPT1A | 8.70E-06 | 2.40E-06 | 0.8 | 0.0002 |
| Phosphorous | 9 | cg06690548 | 4 | 139162808 | SLC7A11 | 2.90E-05 | 8.00E-06 | 0.8 | 0.0003 |
| Alcohol servings | 10 | cg06690548 | 4 | 139162808 | SLC7A11 | -0.0037 | 0.0002 | 14.4 | 3.40E-58 |
| Alcohol | 10 | cg06690548 | 4 | 139162808 | SLC7A11 | -0.0019 | 0.0001 | 14.3 | 1.85E-57 |
| Alcohol servings | 10 | cg14476101 | 1 | 120255992 | PHGDH | -0.0024 | 0.0002 | 7 | 8.26E-28 |
| Alcohol | 10 | cg14476101 | 1 | 120255992 | PHGDH | -0.0013 | 0.0001 | 6.9 | 8.34E-28 |
| Alcohol | 10 | cg18120259 | 6 | 43894639 | LOC100132354 | -0.0007 | 0.0001 | 6.4 | 9.08E-26 |
| Alcohol servings | 10 | cg18120259 | 6 | 43894639 | LOC100132354 | -0.0013 | 0.0001 | 6.3 | 1.90E-25 |
| Beer | 10 | cg06690548 | 4 | 139162808 | SLC7A11 | -0.0034 | 0.0004 | 3.7 | 2.21E-15 |
| Alcohol | 10 | cg21429551 | 7 | 30635762 | GARS | -0.0008 | 0.0001 | 3 | 7.88E-13 |

Model adjusted for age, sex, physical activity, smoking, total energy intake, cell-type heterogeneity, and kinship. Sample size: a=1656, b=1669,

Supplementary Table 1 (continued). Dietary and lifestyle factors associated with Homeostatic Model Assessment for Insulin Resistance-associated epigenetic signatures in the Framingham Offspring Study (n = 1655 - 1671)

| **Dietary/lifestyle** | **Diet/lifestyle cluster** | DNA-MS | **Chromosome** | **Position GRCh37/hg19** | **Gene** | **β** | **SE** | **Variance Explained (%)** | **P value** |
| --- | --- | --- | --- | --- | --- | --- | --- | --- | --- |
| Alcohol servings | 10 | cg21429551 | 7 | 30635762 | GARS | -0.0016 | 0.0002 | 3 | 1.02E-12 |
| Beer | 10 | cg14476101 | 1 | 120255992 | PHGDH | -0.0026 | 0.0004 | 2.3 | 4.10E-10 |
| Alcohol | 10 | cg19390658 | 7 | 30636176 | GARS | -0.0006 | 0.0001 | 2 | 6.06E-09 |
| Alcohol servings | 10 | cg19390658 | 7 | 30636176 | GARS | -0.0011 | 0.0002 | 2 | 6.41E-09 |
| Beer | 10 | cg18120259 | 6 | 43894639 | LOC100132354 | -0.0013 | 0.0002 | 2 | 6.64E-09 |
| Alcohol | 10 | cg17058475 | 11 | 68607737 | CPT1A | -0.0002 | 3.60E-05 | 1.8 | 2.63E-08 |
| Alcohol servings | 10 | cg17058475 | 11 | 68607737 | CPT1A | -0.0004 | 6.90E-05 | 1.8 | 4.40E-08 |
| Alcohol servings | 10 | cg13847322 | 9 | 137272766 | RXRA | -0.0006 | 0.0001 | 1.5 | 8.48E-07 |
| Alcohol | 10 | cg13847322 | 9 | 137272766 | RXRA | -0.0003 | 0.0001 | 1.4 | 1.56E-06 |
| Alcohol | 10 | cg00574958 | 11 | 68607622 | CPT1A | -0.0001 | 2.80E-05 | 1.3 | 2.85E-06 |
| Alcohol servings | 10 | cg00574958 | 11 | 68607622 | CPT1A | -0.0002 | 5.30E-05 | 1.3 | 3.45E-06 |
| Beer | 10 | cg21429551 | 7 | 30635762 | GARS | -0.0019 | 0.0004 | 1.3 | 3.57E-06 |
| Manganese without supplements | 11 | cg06690548 | 4 | 139162808 | SLC7A11 | 0.0082 | 0.0017 | 1.4 | 1.52E-06 |
| AOAC fiber | 11 | cg00574958 | 11 | 68607622 | CPT1A | 0.0003 | 0.0001 | 1.3 | 4.87E-06 |
| Magnesium without supplements | 11 | cg00574958 | 11 | 68607622 | CPT1A | 3.10E-05 | 7.10E-06 | 1.1 | 1.30E-05 |
| AOAC fiber | 11 | cg06690548 | 4 | 139162808 | SLC7A11 | 0.0014 | 0.0003 | 1.1 | 1.49E-05 |
| Potassium | 11 | cg00574958 | 11 | 68607622 | CPT1A | 3.00E-06 | 6.90E-07 | 1.1 | 1.54E-05 |
| Potassium without supplements | 11 | cg00574958 | 11 | 68607622 | CPT1A | 3.00E-06 | 6.90E-07 | 1.1 | 1.62E-05 |
| Potassium | 11 | cg17058475 | 11 | 68607737 | CPT1A | 3.92E-06 | 9.10E-07 | 1.1 | 1.65E-05 |
| Potassium without supplements | 11 | cg17058475 | 11 | 68607737 | CPT1A | 3.82E-06 | 9.10E-07 | 1 | 2.93E-05 |
| Copper without supplements | 11 | cg06690548 | 4 | 139162808 | SLC7A11 | 0.0206 | 0.0053 | 0.9 | 0.0001 |
| AOAC fiber | 11 | cg21429551 | 7 | 30635762 | GARS | 0.0012 | 0.0003 | 0.9 | 0.0001 |
| Manganese without supplements | 11 | cg00574958 | 11 | 68607622 | CPT1A | 0.0014 | 0.0004 | 0.8 | 0.0002 |
| Manganese without supplements | 11 | cg00574958 | 11 | 68607622 | CPT1A | 0.0014 | 0.0004 | 0.8 | 0.0002 |
| Cholesterol | 12 | cg07504977 | 10 | 102131012 | NA | 0.0001 | 1.20E-05 | 1.4 | 1.36E-06 |
| Arachadonic fatty acid | 12 | cg07504977 | 10 | 102131012 | NA | 0.0774 | 0.0202 | 0.9 | 0.0001 |

Model adjusted for age, sex, physical activity, smoking, total energy intake, cell-type heterogeneity, and kinship

Supplementary Table 1 (continued). Dietary and lifestyle factors associated with Homeostatic Model Assessment for Insulin Resistance-associated epigenetic signatures in the Framingham Offspring Study (n = 1655 - 1671)

| Dietary/lifestyle | Diet/lifestyle cluster | DNA-MS | Chromosome | Position GRCh37/hg19 | Gene | β | SE | Variance Explained (%) | P value |
| --- | --- | --- | --- | --- | --- | --- | --- | --- | --- |
| Animal Protein | 12 | cg07504977 | 10 | 102131012 | NA | 0.0003 | 6.90E-05 | 0.9 | 0.0002 |
| Choline from Sphingomyelin | 12 | cg07504977 | 10 | 102131012 | NA | 0.0007 | 0.0002 | 0.8 | 0.0002 |
| Thiamine B1 without vitamin pills | 16 | cg00574958 | 11 | 68607622 | CPT1A | 0.0054 | 0.001 | 1.6 | 1.91E-07 |
| Riboflavin B2 without vitamin pills | 16 | cg00574958 | 11 | 68607622 | CPT1A | 0.0035 | 0.0007 | 1.5 | 8.48E-07 |
| Thiamine B1 without vitamin pills | 16 | cg17058475 | 11 | 68607737 | CPT1A | 0.0061 | 0.0014 | 1.2 | 6.09E-06 |
| Total Folate without vitamin pills, includes fortified foods | 16 | cg17058475 | 11 | 68607737 | CPT1A | 1.70E-05 | 3.90E-06 | 1.1 | 1.40E-05 |
| Riboflavin B2 without vitamin pills | 16 | cg17058475 | 11 | 68607737 | CPT1A | 0.004 | 0.0009 | 1.1 | 1.82E-05 |
| Thiamine B1 without vitamin pills | 16 | cg06690548 | 4 | 139162808 | SLC7A11 | 0.0176 | 0.0046 | 0.9 | 0.0001 |
| Total Folate without vitamin pills, includes fortified foods | 16 | cg13123009 | 6 | 31681882 | LY6G6E;LY6G6D | -2.10E-05 | 5.50E-06 | 0.8 | 0.0002 |
| Free Choline without supplements | 19 | cg06690548 | 4 | 139162808 | SLC7A11 | -0.0007 | 0.0001 | 1.8 | 4.09E-08 |
| Free Choline, choline-contributing metabolite | 19 | cg06690548 | 4 | 139162808 | SLC7A11 | -0.0005 | 0.0001 | 1.3 | 3.11E-06 |
| Free Choline without supplements | 19 | cg18120259 | 6 | 43894639 | LOC100132354 | -0.0003 | 0.0001 | 1.1 | 1.88E-05 |
| Free Choline, choline-contributing metabolite | 19 | cg14476101 | 1 | 120255992 | PHGDH | -0.0004 | 0.0001 | 0.9 | 0.0001 |
| Free Choline without supplements | 19 | cg14476101 | 1 | 120255992 | PHGDH | -0.0004 | 0.0001 | 0.9 | 0.0002 |
| Natural Bran | 20 | cg06690548 | 4 | 139162808 | SLC7A11 | 0.002 | 0.0005 | 1.2 | 8.41E-06 |
| Bran Score | 20 | cg18120259 | 6 | 43894639 | LOC100132354 | 0.0008 | 0.0002 | 1.2 | 1.1E-05 |
| Whole Grain Amount | 20 | cg06690548 | 4 | 139162808 | SLC7A11 | 0.0004 | 0.0001 | 1.1 | 1.4E-05 |
| Bran Score | 20 | cg06690548 | 4 | 139162808 | SLC7A11 | 0.0015 | 0.0004 | 1.1 | 1.8E-05 |
| Bran Score | 20 | cg14476101 | 1 | 120255992 | PHGDH | 0.0014 | 0.0003 | 1 | 3E-05 |
| Whole Grain Score without Added Bran and Germ | 20 | cg06690548 | 4 | 139162808 | SLC7A11 | 0.0004 | 0.0001 | 1 | 3.1E-05 |
| Whole Grain Amount | 20 | cg18120259 | 6 | 43894639 | LOC100132354 | 0.0002 | 4.90E-05 | 0.9 | 0.0002 |
| Natural Bran | 20 | cg18120259 | 6 | 43894639 | LOC100132354 | 0.0009 | 0.0002 | 0.8 | 0.0002 |
| Bran Score | 20 | cg00574958 | 11 | 68607622 | CPT1A | 0.0003 | 0.0001 | 0.8 | 0.0003 |
| Calcium | 23 | cg17501210 | 6 | 166970252 | RPS6KA2 | 1.20E-05 | 2.60E-06 | 1.3 | 2.11E-06 |
| Lactose | 24 | cg00574958 | 11 | 68607622 | CPT1A | 0.0002 | 3.90E-05 | 1.6 | 3.11E-07 |
| Reduced fat dairy servings | 24 | cg00574958 | 11 | 68607622 | CPT1A | 0.0003 | 6.90E-05 | 1.4 | 1.10E-06 |

Model adjusted for age, sex, physical activity, smoking, total energy intake, cell-type heterogeneity, and kinship

Supplementary Table 1 (continued). Dietary and lifestyle factors associated with Homeostatic Model Assessment for Insulin Resistance-associated epigenetic signatures in the Framingham Offspring Study (n = 1655 - 1671)

| Dietary/lifestyle | Diet/lifestyle cluster | DNA-MS | Chromosome | Position GRCh37/hg19 | Gene | β | SE | Variance Explained (%) | P value |
| --- | --- | --- | --- | --- | --- | --- | --- | --- | --- |
| Reduced fat dairy servings | 24 | cg00574958 | 11 | 68607622 | CPT1A | 0.0003 | 6.90E-05 | 1.4 | 1.10E-06 |
| Calcium without vitamin pills | 24 | cg00574958 | 11 | 68607622 | CPT1A | 7.50E-06 | 1.50E-06 | 1.4 | 1.37E-06 |
| Choline from Phosphocholine | 24 | cg00574958 | 11 | 68607622 | CPT1A | 0.0004 | 8.80E-05 | 1.2 | 9.52E-06 |
| Choline from Glycerophosphocholine | 24 | cg00574958 | 11 | 68607622 | CPT1A | 0.0001 | 2.10E-05 | 1 | 3.5E-05 |
| Skim milk | 24 | cg00574958 | 11 | 68607622 | CPT1A | 0.0003 | 8.20E-05 | 1 | 5.6E-05 |
| Choline from Phosphocholine | 24 | cg17058475 | 11 | 68607737 | CPT1A | 0.0005 | 0.0001 | 0.9 | 7E-05 |
| Dairy Vitamin D | 24 | cg00574958 | 11 | 68607622 | CPT1A | 2.10E-05 | 5.40E-06 | 0.9 | 7.6E-05 |
| Dairy Calcium | 24 | cg00574958 | 11 | 68607622 | CPT1A | 6.20E-06 | 1.60E-06 | 0.9 | 8.9E-05 |
| Calcium without vitamin pills | 24 | cg17058475 | 11 | 68607737 | CPT1A | 7.50E-06 | 2.00E-06 | 0.8 | 0.00023 |
| Reduced fat dairy servings | 24 | cg17058475 | 11 | 68607737 | CPT1A | 0.0003 | 9.20E-05 | 0.8 | 0.00025 |
| Carbohydrates | 25 | cg06690548 | 4 | 139162808 | SLC7A11 | 0.0004 | 4.50E-05 | 3.6 | 4.16E-15 |
| Sucrose | 25 | cg06690548 | 4 | 139162808 | SLC7A11 | 0.0007 | 0.0001 | 3 | 8.07E-13 |
| Glycemic Load | 25 | cg06690548 | 4 | 139162808 | SLC7A11 | 0.0005 | 0.0001 | 2.6 | 3.49E-11 |
| Total Sugars | 25 | cg06690548 | 4 | 139162808 | SLC7A11 | 0.0003 | 0.0001 | 2.4 | 2.16E-10 |
| Carbohydrates | 25 | cg18120259 | 6 | 43894639 | LOC100132354 | 0.0002 | 2.40E-05 | 2.4 | 2.47E-10 |
| Carbohydrates | 25 | cg00574958 | 11 | 68607622 | CPT1A | 0.0001 | 1.00E-05 | 2.1 | 3.07E-09 |
| Glycemic Load | 25 | cg18120259 | 6 | 43894639 | LOC100132354 | 0.0002 | 3.90E-05 | 2 | 5.65E-09 |
| Carbohydrates | 25 | cg14476101 | 1 | 120255992 | PHGDH | 0.0002 | 4.30E-05 | 1.7 | 6.39E-08 |
| Carbohydrates | 25 | cg07504977 | 10 | 102131012 | NA | -0.0001 | 2.80E-05 | 1.6 | 2.10E-07 |
| Carbohydrates | 25 | cg17058475 | 11 | 68607737 | CPT1A | 0.0001 | 1.40E-05 | 1.6 | 3.51E-07 |
| Total Sugars | 25 | cg00574958 | 11 | 68607622 | CPT1A | 0.0001 | 1.20E-05 | 1.5 | 4.75E-07 |
| Sucrose | 25 | cg18120259 | 6 | 43894639 | LOC100132354 | 0.0003 | 0.0001 | 1.5 | 6.78E-07 |
| Total Sugars | 25 | cg18120259 | 6 | 43894639 | LOC100132354 | 0.0001 | 2.70E-05 | 1.4 | 1.82E-06 |
| Glycemic Load | 25 | cg14476101 | 1 | 120255992 | PHGDH | 0.0003 | 0.0001 | 1.3 | 2.94E-06 |
| Total Sugars | 25 | cg17058475 | 11 | 68607737 | CPT1A | 0.0001 | 1.60E-05 | 1.3 | 3.34E-06 |
| Glycemic Load | 25 | cg07504977 | 10 | 102131012 | NA | -0.0002 | 4.60E-05 | 1.3 | 4.07E-06 |
| Sucrose | 25 | cg14476101 | 1 | 120255992 | PHGDH | 0.0005 | 0.0001 | 1.3 | 4.44E-06 |
| Sucrose | 25 | cg06500161 | 21 | 43656587 | ABCG1 | -0.0002 | 5.20E-05 | 1.2 | 5.02E-06 |
| Sucrose | 25 | cg07504977 | 10 | 102131012 | NA | -0.0003 | 6.40E-05 | 1.2 | 5.73E-06 |
| Glycemic Load | 25 | cg00574958 | 11 | 68607622 | CPT1A | 0.0001 | 1.70E-05 | 1.2 | 7.03E-06 |
| Total Sugars | 25 | cg06500161 | 21 | 43656587 | ABCG1 | -0.0001 | 2.60E-05 | 1.1 | 1.9E-05 |
| Carbohydrates | 25 | cg06500161 | 21 | 43656587 | ABCG1 | -0.0001 | 2.30E-05 | 1.1 | 2.1E-05 |
| Total Sugars | 25 | cg07504977 | 10 | 102131012 | NA | -0.0001 | 3.20E-05 | 1 | 3E-05 |
| Total Sugars | 25 | cg14476101 | 1 | 120255992 | PHGDH | 0.0002 | 4.90E-05 | 1 | 5.7E-05 |
| Glycemic Load | 25 | cg17058475 | 11 | 68607737 | CPT1A | 0.0001 | 2.30E-05 | 1 | 6.1E-05 |

Model adjusted for age, sex, physical activity, smoking, total energy intake, cell-type heterogeneity, and kinship

Supplementary Table 1 (continued). Dietary and lifestyle factors associated with Homeostatic Model Assessment for Insulin Resistance-associated epigenetic signatures in the Framingham Offspring Study (n = 1655 - 1671)

| Dietary/lifestyle | Diet/lifestyle cluster | DNA-MS | Chromosome | Position GRCh37/hg19 | Gene | β | SE | Variance Explained (%) | P value |
| --- | --- | --- | --- | --- | --- | --- | --- | --- | --- |
| Palmitoleic fatty acid | 30 | cg07504977 | 10 | 102131012 | NA | 0.0157 | 0.0027 | 2 | 6.74E-09 |
| Hydroxyproline | 30 | cg07504977 | 10 | 102131012 | NA | 0.0424 | 0.0089 | 1.3 | 2.15E-06 |
| Palmitoleic fatty acid | 30 | cg00574958 | 11 | 68607622 | CPT1A | -0.0041 | 0.001 | 1 | 4.4E-05 |
| Natural Food Folate, 2001 | 32 | cg17058475 | 11 | 68607737 | CPT1A | 2.10E-05 | 5.70E-06 | 0.8 | 0.0002 |
| Niacin | 33 | cg06192883 | 15 | 52554171 | MYO5C | -0.0001 | 3.80E-05 | 0.9 | 0.0002 |
| Fructose | 34 | cg17058475 | 11 | 68607737 | CPT1A | 0.0002 | 5.00E-05 | 1 | 3.1E-05 |
| Glucose | 34 | cg17058475 | 11 | 68607737 | CPT1A | 0.0002 | 5.50E-05 | 0.9 | 9.8E-05 |
| Fructose | 34 | cg00574958 | 11 | 68607622 | CPT1A | 0.0001 | 3.80E-05 | 0.9 | 0.0001 |
| Wheat germ | 36 | cg22761431 | 17 | 7609416 | EFNB3 | -0.0044 | 9.50E-04 | 1.3 | 3.79E-06 |
| Added Germ from wheat | 36 | cg22761431 | 17 | 7609416 | EFNB3 | -0.0034 | 9.30E-04 | 0.8 | 0.0003 |
| Vitamin D without vitamin pills | 39 | cg00574958 | 11 | 68607622 | CPT1A | 1.10E-05 | 2.90E-06 | 0.8 | 0.0003 |
| Red wine | 45 | cg06690548 | 4 | 139162808 | SLC7A11 | -0.0039 | 4.90E-04 | 3.6 | 4.23E-15 |
| Red wine | 45 | cg14476101 | 1 | 120255992 | PHGDH | -0.0023 | 4.70E-04 | 1.4 | 1.08E-06 |
| Proanthocyanidin | 45 | cg06690548 | 4 | 139162808 | SLC7A11 | -0.0005 | 0.0001 | 1 | 2.4E-05 |
| Red wine | 45 | cg18120259 | 6 | 43894639 | LOC100132354 | -0.0011 | 0.0003 | 1 | 4.3E-05 |
| Catechin | 50 | cg06690548 | 4 | 139162808 | SLC7A11 | -0.001 | 0.0002 | 1.7 | 9.66E-08 |
| Catechin | 50 | cg14476101 | 1 | 120255992 | PHGDH | -0.0007 | 0.0002 | 1 | 5.2E-05 |
| Apigenin | 51 | cg06690548 | 4 | 139162808 | SLC7A11 | -0.0083 | 0.0015 | 1.7 | 7.55E-08 |
| Animal fat (energy adjusted) | 58 | cg07504977 | 10 | 102131012 | NA | 0.001 | 0.0002 | 1.5 | 6.81E-07 |
| Animal fat (energy adjusted) | 58 | cg00574958 | 11 | 68607622 | CPT1A | -0.0003 | 0.0001 | 1 | 6.2E-05 |
| Saturated fat (energy adjusted) | 58 | cg00574958 | 11 | 68607622 | CPT1A | -0.0006 | 0.0002 | 0.9 | 0.00011 |
| Carbohydrate (energy adjusted) | 59 | cg06690548 | 4 | 139162808 | SLC7A11 | 0.0018 | 0.0002 | 3.6 | 5.33E-15 |
| Carbohydrate (energy adjusted) | 59 | cg18120259 | 6 | 43894639 | LOC100132354 | 0.0008 | 0.0001 | 2.4 | 1.60E-10 |
| Total Sugars (energy adjusted) | 59 | cg06690548 | 4 | 139162808 | SLC7A11 | 0.0017 | 0.0003 | 2.4 | 1.79E-10 |
| Carbohydrate (energy adjusted) | 59 | cg00574958 | 11 | 68607622 | CPT1A | 0.0003 | 0.0001 | 2.2 | 8.79E-10 |
| Carbohydrate (energy adjusted) | 59 | cg14476101 | 1 | 120255992 | PHGDH | 0.0012 | 0.0002 | 1.9 | 2.34E-08 |
| Total Sugars (energy adjusted) | 59 | cg00574958 | 11 | 68607622 | CPT1A | 0.0003 | 0.0001 | 1.8 | 3.03E-08 |
| Total Sugars (energy adjusted) | 59 | cg18120259 | 6 | 43894639 | LOC100132354 | 0.0007 | 0.0001 | 1.5 | 7.73E-07 |
| Carbohydrate (energy adjusted) | 59 | cg17058475 | 11 | 68607737 | CPT1A | 0.0003 | 0.0001 | 1.4 | 1.15E-06 |
| Carbohydrate (energy adjusted) | 59 | cg07504977 | 10 | 102131012 | NA | -0.0007 | 0.0001 | 1.4 | 1.89E-06 |
| Total Sugars (energy adjusted) | 59 | cg17058475 | 11 | 68607737 | CPT1A | 0.0004 | 0.0001 | 1.3 | 2.94E-06 |
| Carbohydrate (energy adjusted) | 59 | cg06500161 | 21 | 43656587 | ABCG1 | -0.0005 | 0.0001 | 1.1 | 1.9E-05 |

Model adjusted for age, sex, physical activity, smoking, total energy intake, cell-type heterogeneity, and kinship

Supplementary Table 1 (continued). Dietary and lifestyle factors associated with Homeostatic Model Assessment for Insulin Resistance-associated epigenetic signatures in the Framingham Offspring Study (n = 1655 - 1671)

| Dietary/lifestyle | Diet/lifestyle cluster | DNA-MS | Chromosome | Position GRCh37/hg19 | Gene | β | SE | Variance Explained (%) | P value |
| --- | --- | --- | --- | --- | --- | --- | --- | --- | --- |
| Total Sugars (energy adjusted) | 59 | cg14476101 | 1 | 120255992 | PHGDH | 0.0011 | 0.0003 | 1.1 | 2.4E-05 |
| Total Sugars (energy adjusted) | 59 | cg06500161 | 21 | 43656587 | ABCG1 | -0.0006 | 0.0001 | 1 | 3.3E-05 |
| Total Sugars (energy adjusted) | 59 | cg07504977 | 10 | 102131012 | NA | -0.0007 | 0.0002 | 1 | 5.4E-05 |
| Butter | 72 | cg00574958 | 11 | 68607622 | CPT1A | -0.0003 | 0.0001 | 0.9 | 7.3E-05 |
| Processed Meat servings | 105 | cg07504977 | 10 | 102131012 | NA | 0.0017 | 0.0004 | 1 | 6.9E-05 |
| Liver | 106 | cg09581649 | 16 | 4572735 | C16orf5 | -0.0231 | 0.0061 | 0.9 | 0.0002 |
| Brown rice | 117 | cg17901584 | 1 | 55353706 | DHCR24 | 0.0082 | 0.002 | 1.1 | 2.7E-05 |
| Low calorie cola, no caffeine | 126 | cg06808571 | 7 | 150642256 | KCNH2 | 0.001 | 0.0002 | 1 | 3.2E-05 |
| White wine | 132 | cg06690548 | 4 | 139162808 | SLC7A11 | -0.0051 | 0.0006 | 4.3 | 9.14E-18 |
| White wine | 132 | cg14476101 | 1 | 120255992 | PHGDH | -0.0029 | 0.0006 | 1.5 | 3.92E-07 |
| White wine | 132 | cg18120259 | 6 | 43894639 | LOC100132354 | -0.0014 | 0.0003 | 1.2 | 9.11E-06 |
| White wine | 132 | cg12593793 | 1 | 156074135 | NA | 0.0009 | 0.0002 | 0.8 | 0.0003 |
| Liquor | 133 | cg06690548 | 4 | 139162808 | SLC7A11 | -0.0045 | 0.0005 | 4.9 | 8.98E-20 |
| Liquor | 133 | cg18120259 | 6 | 43894639 | LOC100132354 | -0.0019 | 0.0003 | 3.3 | 8.58E-14 |
| Liquor | 133 | cg14476101 | 1 | 120255992 | PHGDH | -0.003 | 0.0005 | 2.5 | 6.36E-11 |
| Liquor | 133 | cg17058475 | 11 | 68607737 | CPT1A | -0.0008 | 0.0001 | 1.6 | 1.90E-07 |
| Liquor | 133 | cg19390658 | 7 | 30636176 | GARS | -0.0018 | 0.0004 | 1.2 | 5.56E-06 |
| Liquor | 133 | cg21429551 | 7 | 30635762 | GARS | -0.002 | 0.0005 | 1.1 | 2.2E-05 |
| Liquor | 133 | cg07504977 | 10 | 102131012 | NA | 0.0013 | 0.0003 | 1.1 | 2.2E-05 |
| Liquor | 133 | cg00574958 | 11 | 68607622 | CPT1A | -0.0004 | 0.0001 | 0.8 | 0.00019 |
| Sweet baked goods servings | 138 | cg06690548 | 4 | 139162808 | SLC7A11 | 0.0017 | 0.0003 | 1.5 | 5.41E-07 |
| Mustard | 151 | cg18120259 | 6 | 43894639 | LOC100132354 | -0.0023 | 0.0006 | 0.9 | 0.0001 |
| DASH diet | 157 | cg00574958 | 11 | 68607622 | CPT1A | 0.0003 | 8.40E-05 | 0.9 | 9.2E-05 |
| DASH diet | 157 | cg21429551 | 7 | 30635762 | GARS | 0.0014 | 0.0004 | 0.9 | 0.0001 |
| DASH diet | 157 | cg06690548 | 4 | 139162808 | SLC7A11 | 0.0014 | 0.0004 | 0.8 | 0.0002 |
| Jams/jellies | 170 | cg12593793 | 1 | 156074135 | NA | 0.0009 | 0.0003 | 0.8 | 0.0002 |
| Jams & Jellies servings | 170 | cg12593793 | 1 | 156074135 | NA | 0.0009 | 0.0003 | 0.8 | 0.0003 |

Model adjusted for age, sex, physical activity, smoking, total energy intake, cell-type heterogeneity, and kinship

Supplementary Table 2. Direct and indirect effects of dietary/lifestyle factors on Homeostatic Model Assessment for Insulin Resistance through epigenetic signatures in the 8th exam Framingham Offspring Study

| DNA-MS | Chromosome | Position | Genes | DNA-MS cluster | Dietary/lifestyle | Effect | β | SE | *P* value |
| --- | --- | --- | --- | --- | --- | --- | --- | --- | --- |
| cg00574958 | 11 | 68607622 | CPT1A | 8 | Thiamine B1 without vitamin pills | TE | -0.1616 | 0.0385 | <.0001 |
| cg00574958 | 11 | 68607622 | CPT1A | 8 | Thiamine B1 without vitamin pills | NDE | -0.1383 | 0.0390 | 0.0004 |
| cg00574958 | 11 | 68607622 | CPT1A | 8 | Thiamine B1 without vitamin pills | NIE | -0.0233 | 0.0071 | 0.0011 |
| cg00574958 | 11 | 68607622 | CPT1A | 8 | Lactose | TE | -0.0014 | 0.0014 | 0.3128 |
| cg00574958 | 11 | 68607622 | CPT1A | 8 | Lactose | NDE | -0.0004 | 0.0014 | 0.8006 |
| cg00574958 | 11 | 68607622 | CPT1A | 8 | Lactose | NIE | -0.0011 | 0.0003 | 0.0001 |
| cg00574958 | 11 | 68607622 | CPT1A | 8 | Phosphorous | TE | -0.0002 | . |  |
| cg00574958 | 11 | 68607622 | CPT1A | 8 | Phosphorous | NDE | -0.0001 | . |  |
| cg00574958 | 11 | 68607622 | CPT1A | 8 | Phosphorous | NIE | -0.00004 | . |  |
| cg00574958 | 11 | 68607622 | CPT1A | 8 | Total fat (energy adjusted) | TE | -0.0216 | 0.0250 | 0.3882 |
| cg00574958 | 11 | 68607622 | CPT1A | 8 | Total fat (energy adjusted) | NDE | -0.0129 | 0.0253 | 0.6093 |
| cg00574958 | 11 | 68607622 | CPT1A | 8 | Total fat (energy adjusted) | NIE | -0.0087 | 0.0025 | 0.0007 |
| cg06690548 | 4 | 139162808 | SLC7A11 | 4 | Red wine | TE | -0.0098 | 0.0046 | 0.0345 |
| cg06690548 | 4 | 139162808 | SLC7A11 | 4 | Red wine | NDE | -0.0151 | 0.0046 | 0.0011 |
| cg06690548 | 4 | 139162808 | SLC7A11 | 4 | Red wine | NIE | 0.0053 | 0.0010 | <.0001 |
| cg06690548 | 4 | 139162808 | SLC7A11 | 4 | White wine | TE | -0.0139 | 0.0057 | 0.0147 |
| cg06690548 | 4 | 139162808 | SLC7A11 | 4 | White wine | NDE | -0.0205 | 0.0057 | 0.0003 |
| cg06690548 | 4 | 139162808 | SLC7A11 | 4 | White wine | NIE | 0.0066 | 0.0013 | <.0001 |
| cg06690548 | 4 | 139162808 | SLC7A11 | 4 | Liquor | TE | -0.0029 | 0.0044 | 0.5096 |
| cg06690548 | 4 | 139162808 | SLC7A11 | 4 | Liquor | NDE | -0.0087 | 0.0044 | 0.0473 |
| cg06690548 | 4 | 139162808 | SLC7A11 | 4 | Liquor | NIE | 0.0058 | 0.0011 | <.0001 |
| cg06690548 | 4 | 139162808 | SLC7A11 | 4 | Sweet baked goods servings | TE | -0.0006 | 0.0028 | 0.8193 |
| cg06690548 | 4 | 139162808 | SLC7A11 | 4 | Sweet baked goods servings | NDE | 0.0016 | 0.0028 | 0.5623 |
| cg06690548 | 4 | 139162808 | SLC7A11 | 4 | Sweet baked goods servings | NIE | -0.0023 | 0.0006 | <.0001 |
| cg06690548 | 4 | 139162808 | SLC7A11 | 4 | Alcohol servings | TE | -0.0054 | 0.0022 | 0.012 |
| cg06690548 | 4 | 139162808 | SLC7A11 | 4 | Alcohol servings | NDE | -0.0112 | 0.0022 | <.0001 |
| cg06690548 | 4 | 139162808 | SLC7A11 | 4 | Alcohol servings | NIE | 0.0058 | 0.0009 | <.0001 |

Model adjusted for age, sex, physical activity, smoking, total energy intake, cell-type heterogeneity, and kinship

TE = Total effect, NDE = Natural direct effect, NIE = Natural indirect effect

Supplementary Table 2 (continued). Direct and indirect effects of dietary/lifestyle factors on Homeostatic Model Assessment for Insulin Resistance through epigenetic signatures in the 8th exam Framingham Offspring Study

| DNA-MS | Chromosome | Position | Genes | DNA-MS cluster | Dietary/lifestyle | Effect | β | SE | *P* value |
| --- | --- | --- | --- | --- | --- | --- | --- | --- | --- |
| cg06690548 | 4 | 139162808 | SLC7A11 | 4 | Natural Bran | TE | -0.0029 | 0.0038 | 0.4441 |
| cg06690548 | 4 | 139162808 | SLC7A11 | 4 | Natural Bran | NDE | 0.0004 | 0.0038 | 0.9223 |
| cg06690548 | 4 | 139162808 | SLC7A11 | 4 | Natural Bran | NIE | -0.0033 | 0.0009 | 0.0002 |
| cg06690548 | 4 | 139162808 | SLC7A11 | 4 | Carbohydrate (energy adjusted) | TE | -0.0051 | 0.0018 | 0.0054 |
| cg06690548 | 4 | 139162808 | SLC7A11 | 4 | Carbohydrate (energy adjusted) | NDE | -0.0028 | 0.0018 | 0.135 |
| cg06690548 | 4 | 139162808 | SLC7A11 | 4 | Carbohydrate (energy adjusted) | NIE | -0.0023 | 0.0005 | <.0001 |
| cg06690548 | 4 | 139162808 | SLC7A11 | 4 | Free Choline without supplements | TE | -0.0048 | 0.0010 | <.0001 |
| cg06690548 | 4 | 139162808 | SLC7A11 | 4 | Free Choline without supplements | NDE | -0.0056 | 0.0010 | <.0001 |
| cg06690548 | 4 | 139162808 | SLC7A11 | 4 | Free Choline without supplements | NIE | 0.0009 | 0.0002 | <.0001 |
| cg06690548 | 4 | 139162808 | SLC7A11 | 4 | Manganese without supplements | TE | -0.0723 | 0.0138 | <.0001 |
| cg06690548 | 4 | 139162808 | SLC7A11 | 4 | Manganese without supplements | NDE | -0.0603 | 0.0138 | <.0001 |
| cg06690548 | 4 | 139162808 | SLC7A11 | 4 | Manganese without supplements | NIE | -0.0119 | 0.0031 | 0.0001 |
| cg06690548 | 4 | 139162808 | SLC7A11 | 4 | Sucrose | TE | -0.0012 | 0.0009 | 0.1581 |
| cg06690548 | 4 | 139162808 | SLC7A11 | 4 | Sucrose | NDE | -0.0002 | 0.0009 | 0.814 |
| cg06690548 | 4 | 139162808 | SLC7A11 | 4 | Sucrose | NIE | -0.0010 | 0.0002 | <.0001 |
| cg06690548 | 4 | 139162808 | SLC7A11 | 4 | Apigenin | TE | -0.0217 | 0.0133 | 0.1016 |
| cg06690548 | 4 | 139162808 | SLC7A11 | 4 | Apigenin | NDE | -0.0326 | 0.0134 | 0.0148 |
| cg06690548 | 4 | 139162808 | SLC7A11 | 4 | Apigenin | NIE | 0.0108 | 0.0026 | <.0001 |
| cg06690548 | 4 | 139162808 | SLC7A11 | 4 | Catechin | TE | -0.0078 | 0.0016 | <.0001 |
| cg06690548 | 4 | 139162808 | SLC7A11 | 4 | Catechin | NDE | -0.0092 | 0.0015 | <.0001 |
| cg06690548 | 4 | 139162808 | SLC7A11 | 4 | Catechin | NIE | 0.0014 | 0.0003 | <.0001 |
| cg06808571 | 7 | 150642256 | KCNH2 | 6 | Low calorie cola, no caffeine | TE | 0.0159 | 0.0052 | 0.0024 |
| cg06808571 | 7 | 150642256 | KCNH2 | 6 | Low calorie cola, no caffeine | NDE | 0.0133 | 0.0052 | 0.0108 |
| cg06808571 | 7 | 150642256 | KCNH2 | 6 | Low calorie cola, no caffeine | NIE | 0.0026 | 0.0008 | 0.0012 |
| cg07504977 | 10 | 102131012 | NA | 7 | Processed Meat servings | TE | 0.0489 | 0.0059 | <.0001 |
| cg07504977 | 10 | 102131012 | NA | 7 | Processed Meat servings | NDE | 0.0454 | 0.0059 | <.0001 |
| cg07504977 | 10 | 102131012 | NA | 7 | Processed Meat servings | NIE | 0.0035 | 0.0010 | 0.0007 |
| cg07504977 | 10 | 102131012 | NA | 7 | Animal fat (energy adjusted) | TE | 0.0184 | 0.0028 | <.0001 |
| cg07504977 | 10 | 102131012 | NA | 7 | Animal fat (energy adjusted) | NDE | 0.0164 | 0.0028 | <.0001 |
| cg07504977 | 10 | 102131012 | NA | 7 | Animal fat (energy adjusted) | NIE | 0.0021 | 0.0005 | 0.0001 |

Model adjusted for age, sex, physical activity, smoking, total energy intake, cell-type heterogeneity, and kinship

TE = Total effect, NDE = Natural direct effect, NIE = Natural indirect effect

Supplementary Table 2 (continued). Direct and indirect effects of dietary/lifestyle factors on Homeostatic Model Assessment for Insulin Resistance through epigenetic signatures in the 8th exam Framingham Offspring Study

| DNA-MSs | Chromosome | Position | Genes | DNA-MS cluster | Dietary/lifestyle | Effect | β | SE | *P* value |
| --- | --- | --- | --- | --- | --- | --- | --- | --- | --- |
| cg07504977 | 10 | 102131012 | NA | 7 | Cholesterol | TE | 0.0004 | 0.0002 | 0.0103 |
| cg07504977 | 10 | 102131012 | NA | 7 | Cholesterol | NDE | 0.0003 | 0.0002 | 0.0684 |
| cg07504977 | 10 | 102131012 | NA | 7 | Cholesterol | NIE | 0.0001 | 0.00003 | 0.0001 |
| cg07504977 | 10 | 102131012 | NA | 7 | c9,t11 conjugated diene isomer 18:2 Linoleic | TE | 0.0025 | 0.0004 | <.0001 |
| cg07504977 | 10 | 102131012 | NA | 7 | c9,t11 conjugated diene isomer 18:2 Linoleic | NDE | 0.0022 | 0.0004 | <.0001 |
| cg07504977 | 10 | 102131012 | NA | 7 | c9,t11 conjugated diene isomer 18:2 Linoleic | NIE | 0.0003 | 0.0001 | 0.0004 |
| cg07504977 | 10 | 102131012 | NA | 7 | Palmitic fatty acid | TE | 0.0354 | 0.0050 | <.0001 |
| cg07504977 | 10 | 102131012 | NA | 7 | Palmitic fatty acid | NDE | 0.0321 | 0.0050 | <.0001 |
| cg07504977 | 10 | 102131012 | NA | 7 | Palmitic fatty acid | NIE | 0.0034 | 0.0010 | 0.0005 |
| cg07504977 | 10 | 102131012 | NA | 7 | Palmitoleic fatty acid | TE | 0.2016 | 0.0364 | <.0001 |
| cg07504977 | 10 | 102131012 | NA | 7 | Palmitoleic fatty acid | NDE | 0.1684 | 0.0369 | <.0001 |
| cg07504977 | 10 | 102131012 | NA | 7 | Palmitoleic fatty acid | NIE | 0.0332 | 0.0085 | <.0001 |
| cg14476101 | 1 | 120255992 | PHGDH | 2 | Dihydovitamin K | TE | 0.0051 | 0.0013 | 0.0001 |
| cg14476101 | 1 | 120255992 | PHGDH | 2 | Dihydovitamin K | NDE | 0.0059 | 0.0013 | <.0001 |
| cg14476101 | 1 | 120255992 | PHGDH | 2 | Dihydovitamin K | NIE | -0.0008 | 0.0002 | 0.001 |
| cg17501210 | 6 | 166970252 | RPS6KA2 | 5 | Calcium | TE | -0.0002 | . |  |
| cg17501210 | 6 | 166970252 | RPS6KA2 | 5 | Calcium | NDE | -0.0001 | . |  |
| cg17501210 | 6 | 166970252 | RPS6KA2 | 5 | Calcium | NIE | -0.00002 | . |  |
| cg17901584 | 1 | 55353706 | DHCR24 | 1 | Brown rice | TE | -0.0500 | 0.0247 | 0.0424 |
| cg17901584 | 1 | 55353706 | DHCR24 | 1 | Brown rice | NDE | -0.0292 | 0.0248 | 0.2386 |
| cg17901584 | 1 | 55353706 | DHCR24 | 1 | Brown rice | NIE | -0.0208 | 0.0057 | 0.0003 |
| cg22761431 | 17 | 7609416 | EFNB3 | 10 | Wheat germ | TE | -0.0216 | 0.0250 | 0.3882 |
| cg22761431 | 17 | 7609416 | EFNB3 | 10 | Wheat germ | NDE | -0.0129 | 0.0253 | 0.6093 |
| cg22761431 | 17 | 7609416 | EFNB3 | 10 | Wheat germ | NIE | -0.0087 | 0.00254 | 0.0007 |

Model adjusted for age, sex, physical activity, smoking, total energy intake, cell-type heterogeneity, and kinship

TE = Total effect, NDE = Natural direct effect, NIE = Natural indirect effect

Supplementary Table 3. Epigenetic markers associated with Homeostatic Model Assessment for Insulin Resistance in the GOLDN Study (n=931-936)

| DNA-MSs | Chromosome | Position | Gene | β | SE | Variance Explained (%) | *P* value |
| --- | --- | --- | --- | --- | --- | --- | --- |
|  |  | GRCh37/hg19 |  |  |  |  |  |
| cg14476101 | 1 | 120255992 | PHGDH | -1.8491 | 0.3525 | 2.88 | 1.93E-07 |
| cg12593793 | 1 | 156074135 | NA | -0.9819 | 0.4603 | 0.49 | 0.0332 |
| cg12620005 | 1 | 51779655 | TTC39A | -1.5533 | 0.7226 | 0.50 | 0.0319 |
| cg06690548 | 4 | 139162808 | SLC7A11 | -1.9153 | 0.5467 | 1.31 | 0.0005 |
| cg26403843 | 5 | 158634085 | RNF145 | 2.0509 | 0.5228 | 1.63 | 9.40E-05 |
| cg17501210 | 6 | 166970252 | RPS6KA2 | -1.9479 | 0.5266 | 1.45 | 0.0002 |
| cg18120259 | 6 | 43894639 | LOC100132354 | -1.5732 | 0.4788 | 1.15 | 0.0011 |
| cg13123009 | 6 | 31681882 | LY6G6E;LY6G6D | 1.9006 | 0.7171 | 0.75 | 0.0082 |
| cg21429551 | 7 | 30635762 | GARS | -1.3211 | 0.3257 | 1.74 | 5.41E-05 |
| cg19390658 | 7 | 30636176 | GARS | -1.1902 | 0.3629 | 1.15 | 0.0011 |
| cg07504977 | 10 | 102131012 | NA | 1.7349 | 0.4796 | 1.39 | 0.0003 |
| cg00574958 | 11 | 68607622 | CPT1A | -4.1987 | 0.7016 | 3.72 | 3.09E-09 |
| cg17058475 | 11 | 68607737 | CPT1A | -2.2593 | 0.5197 | 2.00 | 1.53E-05 |
| cg06192883 | 15 | 52554171 | MYO5C | 3.5082 | 0.7932 | 2.07 | 1.09E-05 |
| cg03500056 | 16 | 8814507 | ABAT | 1.5124 | 0.5967 | 0.69 | 0.0114 |
| cg11024682 | 17 | 17730094 | SREBF1 | 2.3512 | 0.6475 | 1.40 | 0.0003 |
| cg07952905 | 17 | 27275352 | PHF12 | 1.787 | 0.5818 | 1.01 | 0.0022 |
| cg06500161 | 21 | 43656587 | ABCG1 | 3.2003 | 0.5056 | 4.14 | 3.83E-10 |
| cg27243685 | 21 | 43642366 | ABCG1 | 1.9402 | 0.8081 | 0.62 | 0.0166 |
| cg09349128 | 22 | 50327986 | NA | -2.1432 | 0.5171 | 1.82 | 3.72E-05 |

Model adjusted for age, sex, physical activity, smoking, cell-type heterogeneity, centers, and kinship

Supplementary Table 4. Dietary or lifestyle factors associated with Homeostatic Model Assessment for Insulin Resistance-associated epigenetic signatures in the GOLDN Study (n=941 - 942)

| DNA-MSs | Dietary/lifestyle | β | SE | Variance Explained (%) | *P* value |
| --- | --- | --- | --- | --- | --- |
| cg25001190 | Total fruits | 0.0021 | 0.0007 | 1.00 | 0.0022 |
| cg25217710 | Total fruits | -0.0010 | 0.0005 | 0.46 | 0.0376 |
| cg25758828 | Alcohol, energy adjusted | -0.0004 | 0.0002 | 0.45 | 0.0416 |
| cg03165356 | Alcohol, energy adjusted | 0.0010 | 0.0004 | 0.85 | 0.0048 |
| cg03165356 | Glycemic load | -0.0001 | 5.4E-05 | 0.59 | 0.0193 |
| cg06690548 | Alcohol, energy adjusted | -0.0009 | 0.0002 | 1.53 | 0.0002 |
| cg06690548 | Carbohydrate, energy adjusted | 0.0002 | 0.0001 | 0.43 | 0.0457 |
| cg13123009 | Alcohol, energy adjusted | 0.0004 | 0.0002 | 0.66 | 0.0133 |
| cg13123009 | Cholesterol | -2.3E-05 | 1.1E-05 | 0.51 | 0.0299 |
| cg18120259 | Alcohol, energy adjusted | -0.0009 | 0.0003 | 1.17 | 0.0010 |
| cg17501210 | Folate | 2.5E-05 | 8.4E-06 | 0.94 | 0.0030 |
| cg17501210 | Carbohydrate, energy adjusted | 0.0004 | 0.0001 | 0.84 | 0.0051 |
| cg17501210 | Glycemic load | 8.0E-05 | 3.5E-05 | 0.55 | 0.0230 |
| cg17501210 | Total fruits | 0.0013 | 0.0006 | 0.51 | 0.0297 |
| cg21429551 | Carbohydrate, energy adjusted | 0.0006 | 0.0002 | 0.87 | 0.0045 |
| cg21429551 | Monounsaturated fatty acid, energy adjusted | -0.0015 | 0.0006 | 0.69 | 0.0110 |
| cg21429551 | Protein, energy adjusted | -0.0015 | 0.0006 | 0.64 | 0.0148 |
| cg21429551 | Cholesterol | -5.3E-05 | 2.3E-05 | 0.55 | 0.0238 |
| cg21429551 | Total fat, energy adjusted | -0.0005 | 0.0003 | 0.45 | 0.0396 |
| cg07504977 | Carbohydrate, energy adjusted | -0.0003 | 0.0001 | 0.62 | 0.0159 |
| cg07504977 | Monounsaturated fatty acid, energy adjusted | 0.0009 | 0.0004 | 0.54 | 0.0243 |
| cg07504977 | Total fat, energy adjusted | 0.0004 | 0.0002 | 0.45 | 0.0397 |
| cg03290131 | Dietary fiber | 0.0004 | 0.0002 | 0.48 | 0.0337 |
| cg00574958 | Carbohydrate, energy adjusted | 0.0004 | 9.1E-05 | 2.07 | 1.0E-05 |
| cg00574958 | Glycemic load | 8.0E-05 | 2.6E-05 | 1.00 | 0.0022 |
| cg00574958 | Alcohol, energy adjusted | -0.0005 | 0.0002 | 0.84 | 0.0050 |
| cg00574958 | Total fat, energy adjusted | -0.0003 | 0.0001 | 0.66 | 0.0128 |
| cg00574958 | Dietary fiber | 0.0003 | 0.0001 | 0.57 | 0.0213 |
| cg00574958 | Monounsaturated fatty acid, energy adjusted | -0.0006 | 0.0003 | 0.55 | 0.0236 |
| cg00574958 | Saturated fatty acid, energy adjusted | -0.0006 | 0.0003 | 0.50 | 0.0317 |

Model adjusted for age, sex, physical activity, smoking, total energy intake, cell-type heterogeneity, centers, and kinship

Supplementary Table 4 (continued). Dietary or lifestyle factors associated with Homeostatic Model Assessment for Insulin Resistance-associated epigenetic signatures in the GOLDN Study (n=941 - 942)

| DNA-MSs | Dietary/lifestyle | β | SE | Variance Explained (%) | *P* value |
| --- | --- | --- | --- | --- | --- |
| cg17058475 | Alcohol, energy adjusted | -0.0007 | 0.0002 | 0.89 | 0.0039 |
| cg17058475 | Carbohydrate, energy adjusted | 0.0003 | 0.0001 | 0.69 | 0.0114 |
| cg17058475 | Glycemic load | 8.3E-05 | 3.5E-05 | 0.59 | 0.0192 |
| cg17058475 | Dietary fiber | 0.0004 | 0.0002 | 0.51 | 0.0291 |
| cg17058475 | Vitamin B12 | -0.0012 | 0.0005 | 0.50 | 0.0309 |
| cg03500056 | Glycemic load | -7.7E-05 | 3.1E-05 | 0.66 | 0.0129 |
| cg11024682 | Glycemic load | -6.1E-05 | 2.8E-05 | 0.49 | 0.0319 |
| cg11024682 | Alcohol, energy adjusted | 0.0004 | 0.0002 | 0.49 | 0.0330 |
| cg07952905 | Vitamin B12 | 0.0011 | 0.0005 | 0.57 | 0.0208 |
| cg27243685 | Folate | 1.3E-05 | 5.5E-06 | 0.57 | 0.0208 |

Model adjusted for age, sex, physical activity, smoking, total energy intake, cell-type heterogeneity, centers, and kinship

Supplementary Table 5. Indirect effects of dietary/lifestyle factors^1^ on Homeostatic Model Assessment for Insulin Resistance through epigenetic signatures in the GOLDN Study

| DNA-MSs | Chromosome | Position | Genes | Dietary/lifestyle | β | SE | *P* value |
| --- | --- | --- | --- | --- | --- | --- | --- |
| cg06690548 | 4 | 139162808 | SLC7A11 | Alcohol, energy adjusted | 0.0015 | 0.0006 | 0.0144 |
| cg07504977 | 10 | 102131012 | NA | Monounsaturated fatty acid, energy adjusted | 0.0002 | 0.0005 | 0.7507 |

^1^Dietary/lifestyle factors similarly examined in the 8th exam Framingham Offspring Study for mediated effects on Homeostatic Model Assessment for Insulin Resistance through epigenetic signatures

Model adjusted for age, sex, physical activity, smoking, total energy intake, cell-type heterogeneity, centers, and kinship
